# Copper-Catalyzed Continuous-Flow Transfer Hydrogenation of Nitroarenes to Anilines: A Scalable and Reliable Protocol

**DOI:** 10.1021/acs.oprd.3c00144

**Published:** 2023-12-21

**Authors:** Katia Martina, Maria Jesus Moran, Maela Manzoli, Mikhail V. Trukhan, Simon Kuhn, Tom Van Gerven, Giancarlo Cravotto

**Affiliations:** † Drug Science and Technology Department and NIS−Interdepartmental Centre for Nanomaterials for Industry and Sustainability, University of Turin, via Pietro Giuria 9, 10125 Turin, Italy; ‡ Department of Chemical Engineering, KU Leuven, Celestijnenlaan 200F, 3001 Leuven, Belgium

**Keywords:** hydrogen transfer, flow chemistry, nitro reduction, copper(0)-supported, anilines

## Abstract

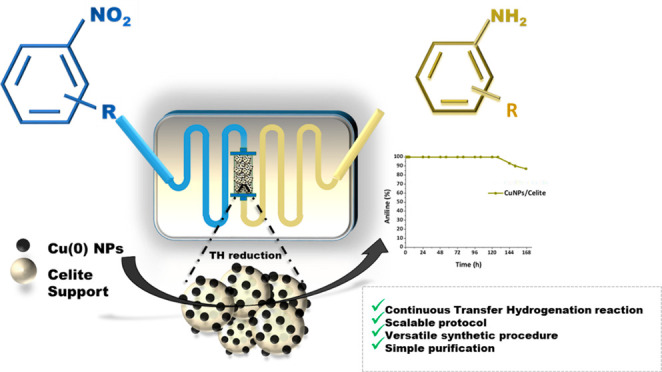

A robust supported catalyst that is made up of copper nanoparticles on Celite has been successfully prepared for the selective transfer hydrogenation of aromatic nitrobenzenes to anilines under continuous flow. The method is efficient and environmentally benign thanks to the absence of hydrogen gas and precious metals. Long-term stability studies show that the catalytic system is able to achieve very high nitrobenzene conversion (>99%) when working for up to 145 h. The versatility of the transfer hydrogenation system has been tested using representative examples of nitroarenes, with moderate-to-excellent yields being obtained. The packed bed reactor (PBR) permits the use of a setup that can provide products via simple isolation by SPE without the need for further purification. The recovery and reuse of either EG or the ion-exchange resin leads to consistent waste reduction; therefore, E-factor distribution analysis has highlighted the environmental efficiency of this synthetic protocol.

## Introduction

Environmental concerns and a desire to face the current planetary emergency have opened the door for an extensive number of green research procedures. Conventional protocols are increasingly being replaced by new efficient synthetic processes that use safer chemicals, naturally abundant solvents, atom economy, and efficient catalytic systems to yield the desired product with sustainability, scalability, and high chemical efficiency. Still to this day, however, conventional technologies in the chemical processing industry tend to be of the batch-type with conduction-based heat-transfer systems and mechanical mixing, which inherently lead to poor process control. Thus, the transition from batch to continuous production is appealing from both sustainability and chemical points of view. Flow approaches have been demonstrated to show great merit in safety and speed, as well as in their increased yields and quality.^1−3^

Hydrogenation reactions are among the most important reactions in pharmaceutical industry API synthesis and make up more than 10% of all chemical transformations.^4^ Precious-metal-catalyzed hydrogenation is the most frequently used procedure, and great effort has been invested in developing appropriate protocols and catalysts for its industrial-scale use.^5−7^ The performance of hydrogenation reactions under flow chemistry has improved considerably in recent times thanks to significant advances in efficiency, safety, and environmental impact.^8,9^ Flow approaches provide better gas–liquid contact than traditional hydrogenation approaches, which are limited by the rate of hydrogen gas diffusion into the bulk solvent.^10,11^ Due to this, flow chemistry is well suited for use in reduction chemistry because of the inherent risks involved in such transformations.^8^ While hydrogen gas or hydride act as the reducing agents in most cases, the in-line production of hydrogen is possible when using smart systems, which also allow mild reaction conditions to be employed.^12,13^ Nevertheless, the use of hydrogen gas is undesirable in industrial applications, as it entails the use of specialized and expensive equipment. Using our experience in transfer hydrogenation approaches for the reduction of nitrobenzene derivatives with ethylene glycol (EG) or glycerol as the hydrogen source,^14,15^ we have striven to develop a completely reliable, fast, safe, and sustainable in-flow procedure for these reactions.

Aniline derivatives are relevant intermediates in the synthesis of dyes, pharmaceuticals, agrochemicals, and other fine compounds, and the reduction of aromatic nitroarenes is clearly the most commonly used method of preparation. Given the importance of this process in both industry and academia, it is not surprising that several catalytic flow-chemistry hydrogenation protocols have been reported over the past decade.^16,17^ The reaction is usually performed under either palladium,^18−21^ platinum^22^ or RANEY nickel,^23,24^ or Ni/SiO_2_^25^ catalysis, giving high yields, and few examples also reported gold,^26^ ruthenium,^27,28^ and cobalt.^29^ All reactions are performed in dedicated high-pressure resistant reactors^30^ at variable temperatures from rt to 110 °C.

Because of advantages in terms of cost and reagent safety, continuous reduction via catalytic hydrogen transfer has also been also reported. In-flow transfer hydrogenation (TH) reactions have already been reported for the reduction of ketones and imines,^31−34^ olefins,^35−37^ and levulinates^38−40^ in order to obtain high-value products, while several studies reported the TH reactions of nitrobenzenes under continuous flow. Based on our knowledge, hydrides have been studied as hydrogen donors in large excess, Au/NaBH_4_^41^ and Pd/NaBH_4_^42^ have been reported in this field, and Pd/C has been also used with a large excess of ammonium formate^43^ and of cyclohexene.^44^ Non-noble metals have also been of great interest to the scientific community due to their economic and environmental advantages, and progress in that regard refers to the use of Fe_3_O_4_/N_2_H_4_^45,46^ Bi–Fe with N_2_H_2_^47^ and a metal-free approach with HSiCl_3_.^48^ Aimed to improve the availability of cost-effective and easily manageable hydrogen donors, alcohols were also studied. Supercritical isopropanol has been employed for the efficient reduction of nitroarenes in a flow-type reactor in the presence of alumina^49^ and zirconia^50^ with short contact times under harsh reaction conditions (545–580 K, 20 MPa), while more recently ethanol was used with Fe/Ru supported on a γ-Al_2_O catalyst (reaction temperature 140 °C).^51^ One example reported an almost full hydrogen utilization and efficiency, and a stoichiometric amount of NH_3_·BH_3_ was used with Pd/C.^52^ Despite progress in this domain, the development of simple, green, efficient, and practical catalytic transfer hydrogenation systems for nitroarene reduction is still highly desirable. In the drive to develop selective catalysts that are able to suppress the use of undesired noble metals, copper has received much attention in recent decades, and its efficient application in catalyzed continuous-flow synthetic processes is reported in the literature.^53−56^ Very few literature refers to Cu-catalyzed continuous hydrogenation,^57−60^ and it focuses on the reduction of 5-HMF; only one example has reported copper-catalyzed transfer hydrogenation in-flow in the presence of a huge excess of NaBH_4_.^61^

As recently reported by our group, copper(0) nanoparticles (CuNPs) are able to catalyze the TH of aromatic nitrobenzenes to anilines and azobenzenes^14,15^ and of alkynes to *cis*-alkenes^62^ in the presence of glycerol or ethylene glycol, where the alcohol acts both as the solvent and hydrogen donor. Inspired by these successful results, the purpose of this study is thus the development of a new continuous-flow procedure for the reduction of nitrobenzenes in the presence of a non-noble, robust, and recyclable supported catalytic Cu system. The study aims to combine the significant benefits attained from heterogeneously catalyzed transfer hydrogenation reactions with translation into scalable continuous-flow chemistry processes. Furthermore, resources that are abundant in chemical industries and that can be produced from renewable biomass, such as glycerol and ethylene glycol, have been exploited for simultaneous use as solvents and the “sacrificial” hydrogen source.

## Experimental Section

All commercially available reagents and solvents were used without further purification. Aluminum oxide, γ-phase 99+%, was purchased from Thermoscientifics, and Celite 545 and activated charcoal was purchased from Sigma-Aldrich. Zeolite 5.1:1, 30:1, and 360:1 (SiO_2_/Al_2_O_3_ ratio) were purchased from Alfa Aesar. The ethylene glycol (EG) used contained no less than 99 w/w % of the principal substance. Reactions were monitored via TLC on Merck 60 F254 (0.25 mm) plates (Milan, Italy), which were visualized using UV inspection and/or by heating after spraying with 0.5% ninhydrin in ethanol. NMR spectra (Jeol ECZ-R 600 and 125 MHz for ^1^H and ^13^C, respectively) were recorded. Chemical shifts were calibrated to the residual proton and carbon resonances of the solvent CDCl_3_ (δH = 7.26, δC = 77.16). Chemical shifts (δ) are given in ppm, and coupling constants (*J*) in Hz. GC conditions were injection split, 1:10; injector temperature, 250 °C; detector temperature, 280 °C; and gas carrier, helium (1.2 mL/min) with a temperature program from 50 °C (5 min) to 100 °C (1 min) at 10 °C/min, to 230 °C (1 min) at 20 °C/min, to 300 °C (5 min) at 20 °C/min. The cations were determined with a PerkinElmer Optima 7000 (PerkinElmer, Norwalk, Connecticut, USA) inductively coupled plasma–optical emission spectrometer (ICP-OES). Six nitrobenzene derivatives (**1**.**6**–**1**.**11**) were synthesized in-house; procedures and characterizations are reported in the Supporting Information.

### General Procedure for the Synthesis of CuNPs

The synthesis was performed as reported in our previous manuscript. Copper(II) sulfate was dissolved in H_2_O/EG (5:1, 0.38 g, 90 mL), and aq NaOH (2 M) was added dropwise to pH = 11. The obtained deep blue solution was stirred vigorously, and aq NaBH_4_ (0.5 M, 5 mL) was added to the flask. Initially, the solution gradually lost color and then turned burgundy, confirming the formation of copper colloids. The CuNPs were recovered on a Büchner funnel and washed with water and methanol.

### General Procedure for Catalyst Synthesis (CuNPs/Support 5 w/w %)

A copper(II) sulfate solution (90 mL of a 26 mM solution in H_2_O/EG 5:1) was stirred, followed by the dropwise addition of a 2 M NaOH aqueous solution, used to adjust the solution pH to 11. The solid support (3 g) was then added. After the mixture was stirred for 10 min, 5 mL of 0.5 M NaBH_4_ in water was slowly added to the flask, while sonication was performed in an ultrasonic bath in order to satisfactorily disperse the particles. The deep blue solution gradually became colorless and then turned burgundy, confirming the formation of copper colloids. The supported copper nanoparticles were filtered on a Büchner funnel with a sintered glass disc, with water and methanol being used to wash the catalyst.

### General Procedure for Catalyst Synthesis (CuNPs + Celite 5 w/w %)

The supported material (Celite 545, 100 mg) and 6 mg of CuNPs (synthesis described in General Procedure for the Synthesis of CuNPs) were physically mixed inside a vial until a homogeneous mixture was obtained. This catalyst will hereafter be denoted CuNPs + Celite.

### Characterization

The CuNPs/Celite was characterized by means of diffuse reflectance (DR) UV–vis-NIR analysis, and powders were placed in a quartz cell, only allowing the spectra to be recorded at room temperature. DR UV–vis-NIR spectra were run on a Varian Cary 5000 spectrophotometer, working in the 190–2500 nm range of wavenumbers. The spectra are reported in accordance with the Kubelka–Munk function: *f*(*R*_*∞*_) = (1 – *R*_*∞*_)^2^/2*R*_*∞*_; *R*_*∞*_ = reflectance of an “infinitely thick” layer of the sample.

All FT-IR spectra were acquired with a Bruker Equinox 55 spectrometer equipped with an MCT detector at a resolution of 4 cm^–1^, averaging 64 scans.

X-ray diffraction (XRD) patterns were collected on a PW3050/60 X’Pert PRO MPD diffractometer from PANalytical, working in Bragg–Brentano geometry using, as a source, a high-powered ceramic tube PW3373/10 LFF with a Cu anode (using Cu Kα1 radiation λ = 1.5406 Å) equipped with a Ni filter to attenuate Kβ. Scattered photons were collected using a real-time multiple strip (RTMS) X’celerator detector. Data were collected in the 10° ≤ 2θ ≤ 100° angular range, with 0.02° 2θ steps. The powdered samples were examined in their as-received form and placed in a spinning sample holder to minimize preferred orientations.

The morphology and composition of the fresh CuNPs/Celite and those after reaction and reactivation were investigated by scanning electron microscopy (SEM; Zeiss Evo50) operating at 20 kV using an energy-dispersive X-ray detector (EDS).

### General Procedure for the Reduction of Nitrobenzene in Batch with Solid-Supported Cu Catalysts

The nitroarene (1 mmol), KOH (2 mmol), and supported CuNPs (63 mg, 5 mol %) were dispersed in 7 mL of either glycerol or EG and heated under magnetic stirring (500 rpm) in an oil bath at 130 °C for either 30 or 60 min. Reaction conversion was monitored by GC-MS.

### General Procedure for Continuous-Flow Transfer Hydrogenation Reduction of Anilines

Experimental procedures were performed in two laboratory-scale setups (setup A and setup B, see the SI for detail and Figures S1–S3) that used a packed bed reactor (PBR) loaded with CuNPs/Celite.

Setup A: The BPR is an in-house-made milliscale reactor in stainless steel with an internal diameter of 0.4 mm with enhanced interfacial mass transfer.^63^ The reactor is 2.6 cm long and is filled with 150 mg of the solid-supported catalyst. The total volume of the reactor (*V*_t_) is 0.327 cm^3^, while the void volume (*V*_0_) is 0.149 cm^3^ (see the SI for *V*_t_ and *R*_v_ measurements, Figure S2).

Setup B: The BPR is the commercially available Syrris Asia flow chemistry system equipped with a glass column reactor 100 mm long. In the present study, 5.6 and 12 mL reactors with adjustable ends have been employed. The BPRs were filled with 250 mg and 1.1, 2.5, and 5 g of CuNPs/Celite, respectively (Figure S3). The reactors’ volumes were *V*_t_ = 0.628 cm^3^ and *V*_0_ = 0.292 for 250 mg; *V*_t_ = 3.412 cm^3^ and *V*_0_ = 0.955 cm^3^ for 1.1 g; *V*_t_ = 7.336 cm^3^ and *V*_0_ = 2.04 cm^3^ for 2.5 g; and *V*_t_ = 14.672 cm^3^ and *V*_0_ = 4.2 cm^3^ for 5.0 g of catalyst.

The nitroaromatic compound (6 mmol) and KOH (12 mmol) were dissolved in 12 mL of EG. The reaction mixture was pumped into the microreactor at the indicated flow rate (mL/min) at room temperature, and the solution was heated to 130 °C by the heating device (either oil bath or Asia Syrris heating block), allowing the reduction reaction to take place. The contact time (τ) was determined as the ratio between the catalyst volume in the reactor *V*_r_ (cm^3^) and the total inlet rate of the mixture (cm^3^/s) (see the SI).

The crude amine solution was purified with a polymeric supported sulfonic resin (Dowex 50 × 8 preliminarily conditioned with HCl 6 N then washed with water and methanol). The crude EG solution was added to a cartridge filled with Dowex 50 × 8 (2 g resin, 10 mL 0.5 M solution resin capacity 6.2 mmol/g), and the resin was left in contact at room temperature for 15 min and filtered. The EG solution recovered was distillated, and the solvent, recovered after NMR analysis to confirm the purity, was reused. The IXR cartridge was washed with 5 mL of ethanol, and this amine-bound resin was treated with 7 mL of a 4 M ammonia methanolic solution at room temperature for 30 min. This resin was filtered and washed with 5 mL of ethanol in order to dissolve all of the desired aniline derivative. The solution was evaporated under vacuum. After usage, the resin was regenerated in HCl 6 N and washed as already reported above.

Products were analyzed using ^1^H NMR and ^13^C NMR spectroscopy and GC-MS chromatography (see the SI).

## Results and Discussion

**Scheme 1:**
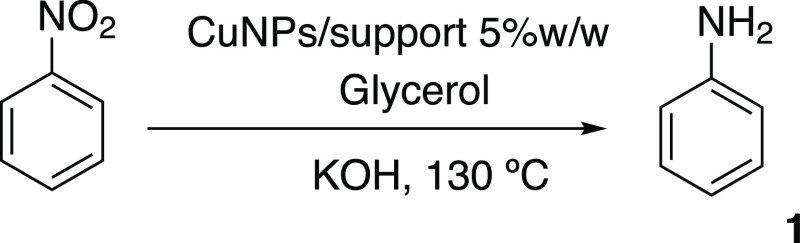
Cu-Catalyzed TH of Nitrobenzene

**Figure 1:**
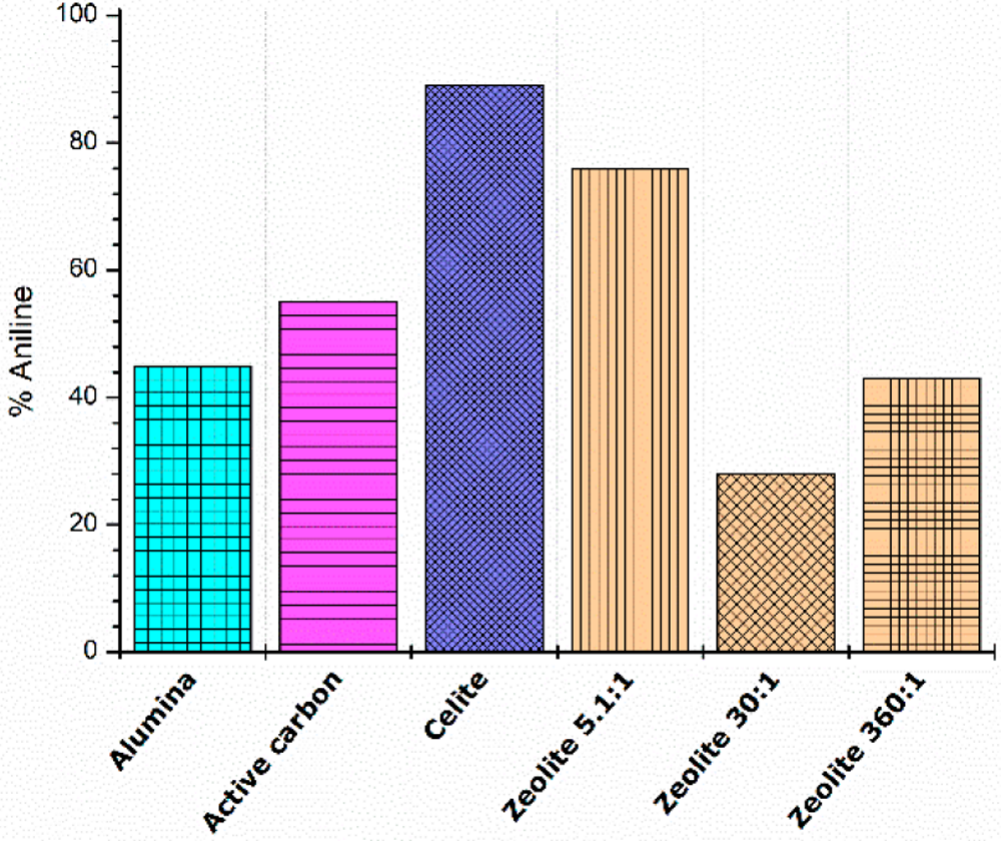
TH of nitrobenzene to aniline in batch in the presence of CuNP catalysts. Reaction conditions: nitrobenzene (1 mmol), KOH (2 mmol), CuNPs/support (5 mol %), and glycerol (7 mL). *T*, 130 °C; *t*, 30 min; magnetic stirring. Conversion (%) determined by GC-MS analysis.

Supported metal catalysts have a high surface-area-to-volume ratio and show comparatively high turnover frequencies,^64^ while their longer durability, the improved reaction reproducibility that they provide, and their recyclability make them preferable to unsupported counterparts.^65^ Several novel methods to immobilize/stabilize active metal species and to separate/collect/reuse them have therefore been proposed.^66^ In order to preliminarily test solid supported Cu(0) nanocatalysts, NP immobilization was performed on a range of solid supports (alumina, charcoal, Celite, zeolite, etc.), and the supported catalysts were then tested in the reduction of nitrobenzene in batch. Based on our previous experience,^14^ the CuNPs were obtained via the reduction of *in situ* prepared Cu(OH)_2_, using NaBH_4_ in water:EG (5:1), and the Cu-supported catalysts were prepared via the deposition–precipitation and activation method with an estimated loading % of 5% w/w Cu(0) NP/support (see the SI). The standard reaction (Scheme 1) between nitrobenzene (**1**.**1**) and KOH was repeated with different catalysts in glycerol at 130 °C for 30 min, and the reaction outcomes were compared. Figure 1 shows the results, and an appreciable difference in behavior was observed. When alumina and active carbon were used, 45% and 55% conversions were obtained, respectively, while in the presence of commercial zeolites, which are known to be able to modulate their acid properties, only zeolite 5.1:1 (SiO_2_:Al_2_O_3_ ratio) gave high conversion, with 80% aniline being achieved. The best result was observed when the Celite-supported catalyst was tested, and fully reduced aniline was recovered in a 90% yield.

**Figure 2:**
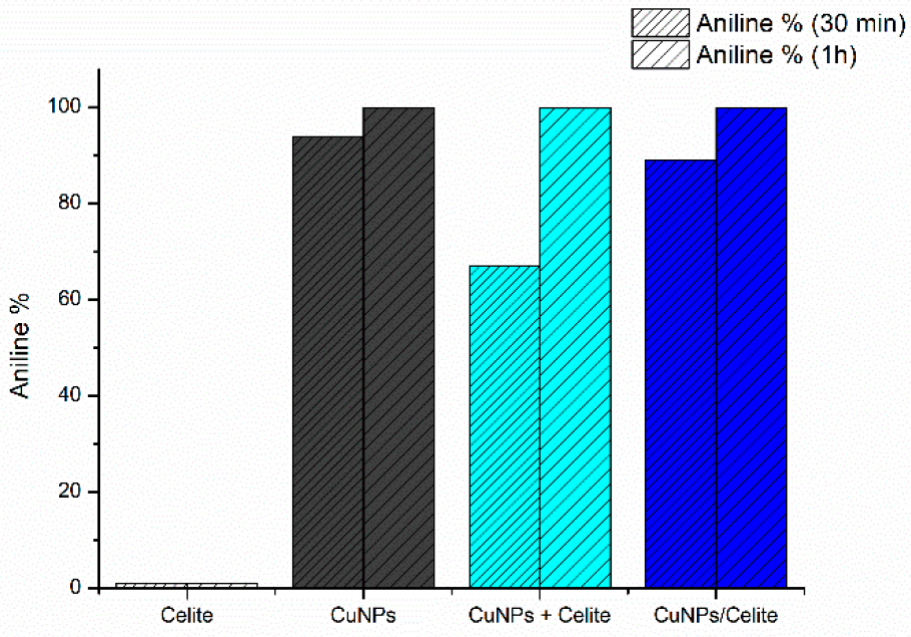
TH of nitrobenzene to aniline in batch in the presence of Celite, CuNPs, CuNPs + Celite, and CuNPs/Celite. Reaction conditions: nitrobenzene (1 mmol), KOH (2 mmol), catalyst (5 mol %), and glycerol (7 mL). *T*, 130 °C; *t*, 30 min and 1 h; magnetic stirring. Conversion determined by GC-MS.

In order to better understand the role of the support, the inorganic Celite, fresh unsupported CuNPs, a physical mixture of the CuNPs and Celite (CuNPs + Celite) and the Celite-supported CuNPs (CuNPs/Celite) were tested under batch conditions and the reaction was monitored after 30 min and 1 h (Figure 2). Because of their higher accessible surface area, unsupported CuNPs presented a slightly higher activity. Regardless, the excellent reactivity of CuNPs/Celite was confirmed. As displayed in Figure 2, the supported material CuNPs/Celite performed better than the physical mixture. The results confirmed the contribution of the solid-supported catalyst to the reaction outcome, and the physical mixture showed slightly diminished reactivity.

**Figure 3:**
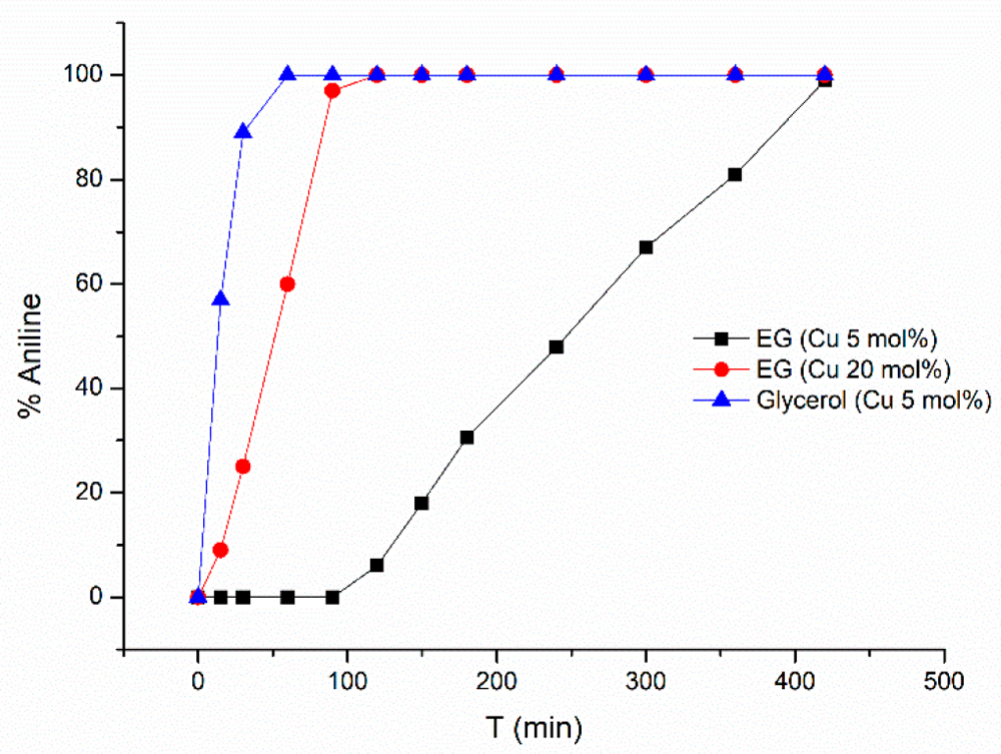
TH kinetics of nitrobenzene reduction. Reaction conditions: nitrobenzene (1 mmol), KOH (2 mmol), catalyst (5 or 20 mol %), and EG or glycerol (7 mL). *T*, 130 °C. Conversion was determined by GC-MS. (▲) Glycerol (Cu 5 mol %), (■) EG (Cu 5 mol %), and (●) EG (Cu 20 mol %).

With an eye to its application in continuous-flow syntheses, the reaction was also performed not only in glycerol but also in ethylene glycol (EG) because its lower viscosity allows flow through the tubes and a small packed bed reactor. As depicted in Figure 3, the reaction conversion to aniline of the experiments was measured over time, and appreciable differences were found between the use of glycerol and EG as reducing agents when both reactions were performed with 5 mol % of solid supported Cu since EG showed a slower reaction rate and the reaction reached complete conversion in approximately 7 h. However, despite this difference, when the reaction was repeated with 20 mol % of Cu-supported catalysts, complete conversion was achieved in 1.5 h in EG. Therefore, we attempted to study the process in continuous flow in EG, trusting that the high efficiency heat and mass transfer of the flow systems, as well as the increased ratio of substrate/catalyst, would lead to a higher reaction rate than the batch version.

**Figure 4:**
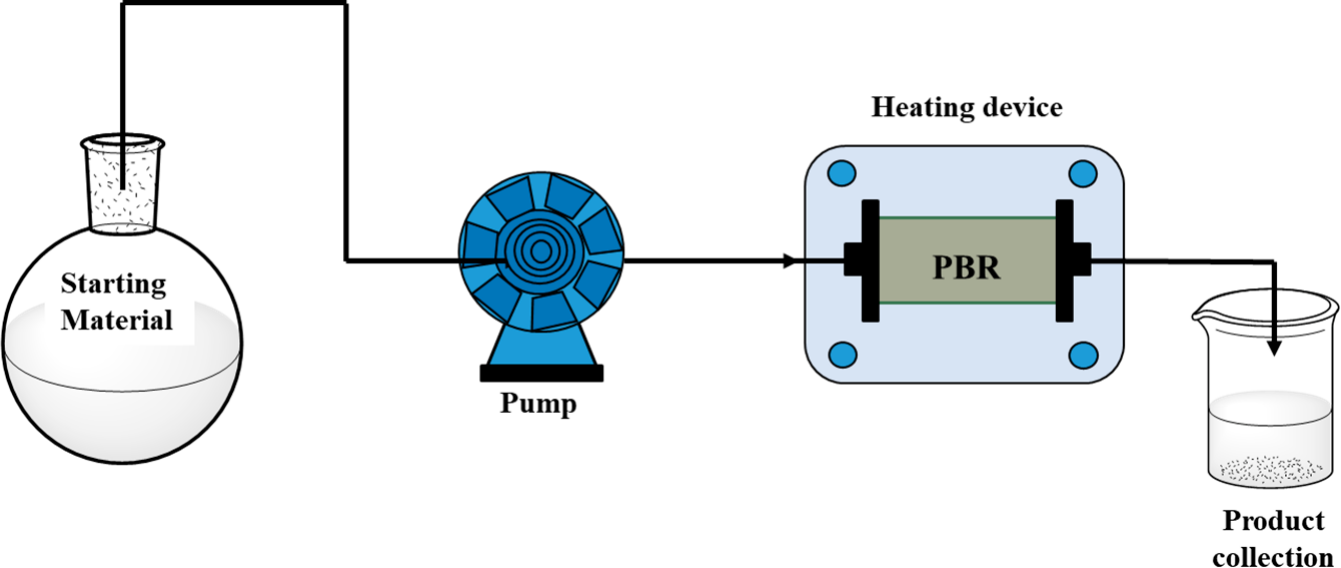
Outline of the apparatus adopted for the continuous test in a packed bed reactor (PBR). Reaction conditions: nitrobenzene (1 mmol), base (2 mmol), hydrogen donor, and CuNPs/Celite.

The catalytic activity of CuNPs/Celite was then investigated under continuous-flow conditions in a packed bed reactor (PBR), thereby allowing the reaction mixture to pass through the catalyst without the need for subsequent removal by filtration. The presence of an excess of catalyst is an additional advantage of PBR, and this results in a significant reduction in the required residence time. The reduction of nitrobenzene to aniline was used as the model reaction; a one-pump setup was designed, and nitrobenzene and KOH, dissolved in a suitable hydrogen donor (0.5 M), were loaded into the sample loop and then pumped through the PBR (see Figure 4). Two different setups were preliminary studied: a setup A composed of an in-house-made milliscale reactor in stainless steel (Ø = 0.4 cm; length = 2.6 cm) filled with 150 mg of the solid supported catalyst (see SI Section 1.2 and Figure S2) and a setup B composed of a commercially available Syrris Asia flow chemistry system equipped with a glass column reactor (Ø = 1 cm) filled with 250 mg of catalyst (length = 0.8 cm; see SI Section 1.2 and Figure S3). The reaction mixture was pumped at the indicated flow rate (mL/min) at room temperature, and the solution was heated to 130 °C when arriving at the heating device. The output of the reactor was collected in a flask and analyzed by GC-MS after six reactor volumes.

Table 1 shows the reaction optimization results obtained under continuous flow using different parameters and two different setups. A smaller-scale optimization was performed using a Harvard syringe pump and the PBR that was heated via the immersion of the reactor inside an oil bath at 130 °C (system 1; see Figure S1A). The PBR was loaded with 150 mg of catalyst, and the TH reduction was optimized by varying several parameters: CuNP content on Celite (5% or 10% w/w), initial nitrobenzene concentration, and flow rate (Table 1). The same experiments were carried out in a second reactor (the Syrris Asia flow chemistry system), in which the PBR was loaded with 250 mg of catalyst (system 2, see Figure S1B). The void volumes of the packed bed reactors were determined for both the cartridges and were found to be 0.149 and 0.293 cm^3^ (about 45% of total volume), respectively (see Figures S2 and S3).

**Table 1:**
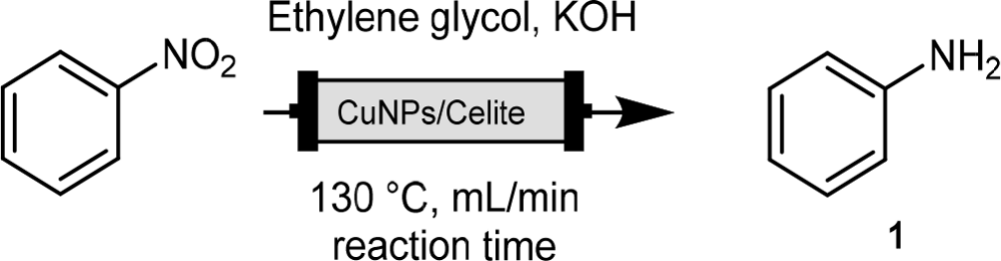
Optimization of Cu-Catalyzed Nitrobenzene Reduction in EG under Continuous Flow

entry^a^	CuNPs/Celite (w/w %)	**1.1** conc. (mol/L)	solvent/hydrogen donor	flow rate (mL/min)	residence time (min)	yield (%)^b^
1	150 mg (10% w/w)	0.5	EG	0.05	2.98^c^	85
2	150 mg (5% w/w)	0.5	EG	0.05	2.98^c^	70
3	250 mg (5% w/w)	0.5	EG/Gly	0.05	5.85^d^	97
4	250 mg (5% w/w)	0.5	EG	0.05	5.85^d^	92
5	250 mg (5% w/w)	0.5	iPrOH	0.03	9.07^d^	22^e^
6	150 mg (10% w/w)	0.5	EG	0.03	4.96^c^	83
7	250 mg (10% w/w)	0.5	EG	0.03	9.07^d^	>99
8	150 mg (5% w/w)	0.5	EG	0.03	4.96^c^	76
9	250 mg (5% w/w)	0.5	EG	0.03	9.07^d^	>99
10	150 mg (10% w/w)	0.5	EG	0.02	7.45^c^	96
11	150 mg (5% w/w)	0.5	EG	0.02	7.45^c^	88
12	150 mg (5% w/w)	0.3	EG	0.02	7.45^c^	92
13	150 mg (5% w/w)	0.1	EG	0.02	7.45^c^	>99

a Reaction performed using 1 mmol of nitrobenzene in EG at the indicated initial concentration and 2 mmol KOH. Reaction temperature: 130 °C.

b Reaction yield determined by GC-MS.

c Setup A: PBR reactor loaded with 150 mg.

d Setup B: PBR reactor loaded with 250 mg.

e Reaction performed in iPrOH at 90 °C. Nitrobenzene:azoxybenzene:aniline, 22:30:48.

Preliminarily, the continuous reaction was attempted in glycerol that has a viscosity of 1499 cP at 20 °C; therefore, we heated the starting solution to 100 °C (21 cP) to perform experiments with both setups A and B. Due to the fact that the temperature of the liquid decreased while the solution passed through tubes and syringes, pumps were blocked for overpressure and we decided to perform the reaction in EG alone, and for sake of comparison, in a mixture of 1:1 EG/Gly. As depicted in Table 1, an efficient and selective reduction of nitrobenzene to aniline was observed when the reaction was performed at a flow rate of 0.05 mL/min in the presence of a BPR filled with CuNPs/Celite 10% and 5% w/w (Table 1, entries 1–4) and reaction conversions increased from 70% to 97% by varying reaction conditions. We could evaluate that the best results were obtained with 250 mg of Cu-supported at 5%, and if compared to 150 mg of Cu-supported at 10%, it was evidenced that increasing the amount of catalyst and the residence time led to better results than increasing the amount of Cu on the catalyst surface (entries 1 and 4 Table 1). The mixture of EG/Gly showed only a slight increase of reaction conversion that was 92% in EG and 97% in the presence of EG/Gly 1:1, thus demonstrating that EG may efficiently act as a hydrogen donor in nitro reduction (Table 1, entry 3). The reaction was therefore performed with setup B (entry 5, Table 1) and catalyzed by 5% CuNPs/Celite (w/w %) at 90 °C in iPrOH. A decrease in product conversion was observed (70%), and the reduction showed a decreased selectivity to aniline: a mixture containing 48% of azobenzene, 30% of nitrobenzene, and 22% of aniline was collected to confirm the higher efficacy of diols to act as hydrogen donors, as already reported when the reaction was performed in batch.^14^ Conversion was complete with 250 mg of catalyst when the flow rate was decreased to 0.03 mL/min (residence time 5.85 min), and no differences were observed with 5% or 10% w/w loaded catalyst (entries 7 and 9, Table 1). When the reaction was performed at 0.02 mL/min flow with 150 mg of catalyst (residence time 7.45 mL/min) we observed 96% or 88% conversion of nitrobenzene to aniline with 5% or 10% w/w loaded catalyst (entries 10 and 11); therefore, we decided to study the influence of the initial nitrobenzene concentration in setup A, and as shown in Table 1 (entries 12 and 13), the higher concentration gave lower nitrobenzene conversion to aniline, whereas quantitative conversion was achieved when the 0.1 M solution was pumped through the 150 mg of CuNPs/Celite 5% w/w PBR at a flow rate of 0.02 mL/min.

**Figure 5:**
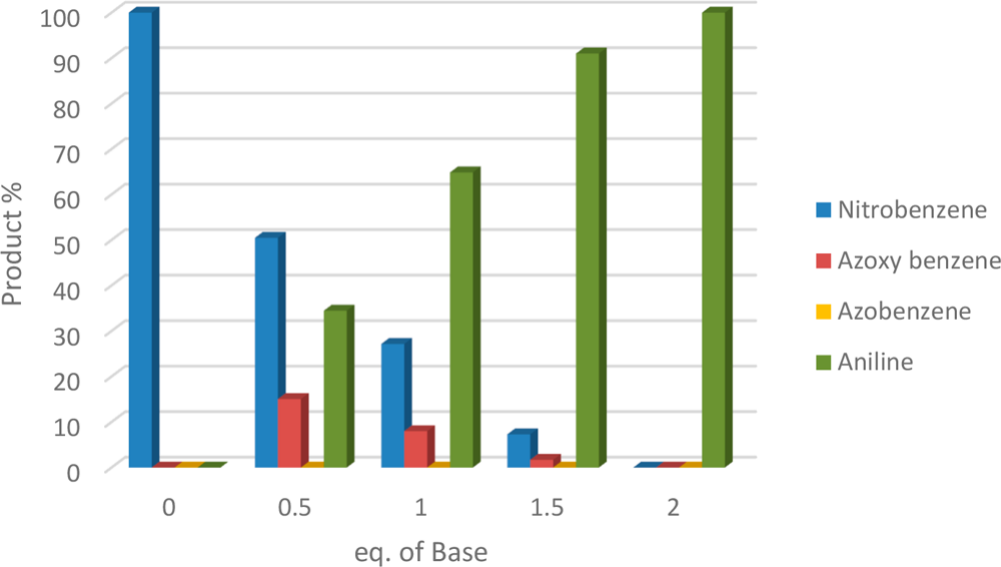
Influence of base equivalents in the nitrobenzene TH. Reaction conditions: nitrobenzene in EG (0.5 M, 1 eq) and KOH. Flow rate: 0.03 mL/min, 250 mg of CuNPs/Celite 5% w/w, setup B (see Figure S1B). Reaction temperature: 130 °C, and the reaction is monitored after 18 reactor volumes. Reaction yield was determined by GC-MS.

It was recognized that in many cases the mixture of base/hydrogen donor (e.g., NaOiPr/iPrOH or NaOtBu/iPrOH) is required to generate the active catalyst from the precatalyst and to accelerate the catalysis beyond the activation step in the TH reaction.^67^ Despite this beneficial effect, the use of a strong base can affect the reaction outcome and product purity when base-sensitive substrates are employed; therefore, we opted for a study aimed to reduce the amount of base. Figure 5 schematically represents results collected from GC-MS analysis of the crude reaction when the reaction was performed with the setup B (250 mg of Cu/Celite 5% w/w) at 0.03 mL/min with an increasing amount of base; not only were nitrobenzene and aniline monitored but also reaction intermediates. In fact, we considered the need to study the product distribution because on the basis of our recent experience with the Cu-catalyzed TH of nitrobenzene in Gly and EG, the condensation route was followed, and not only aniline but also azo and azoxybenzene could be detected. The present study evidenced that in the absence of base, only unreacted nitrobenzene was recovered, while complete conversion and selectivity was obtained with 2 equiv of base. Azoxybenzene was detected in the mixture with nitrobenzene in a percentage from 15% to 2%, passing from 0.5 to 1.5 eq of base to confirm the Haber mechanism via the condensation route (see Figure 5); the conversion of nitrobenzene increased from 51% to >99%.

**Figure 6:**
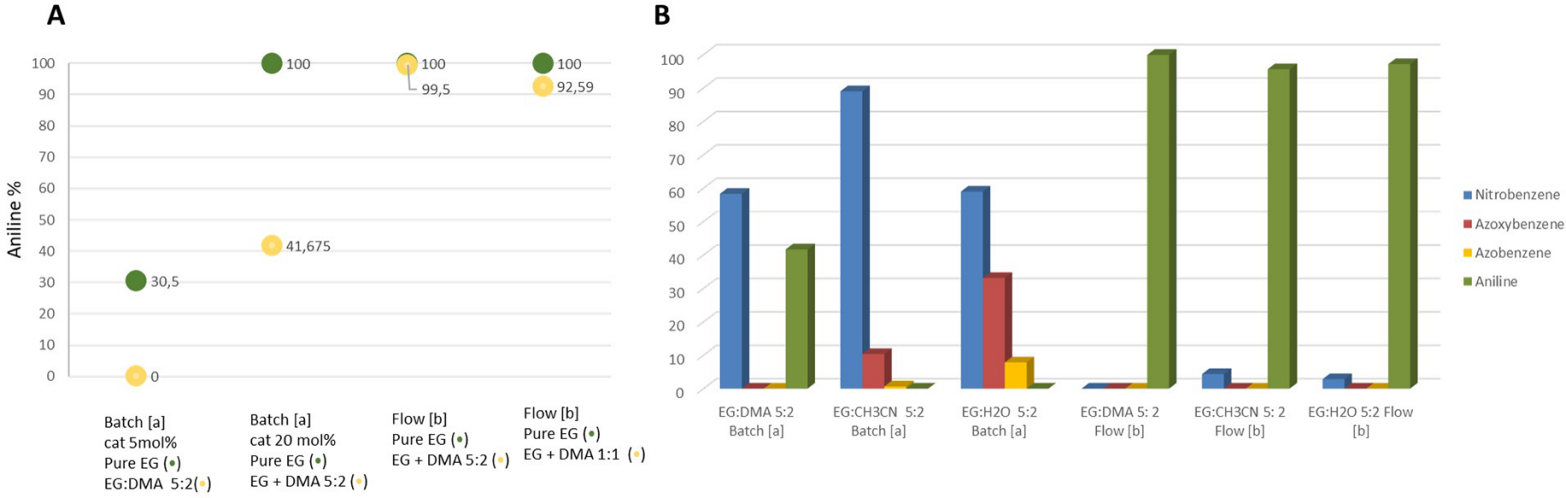
Influence of DMA, CH_3_CN, and water on TH of nitrobenzene. (a) Batch reaction condition: nitrobenzene (2.5 mmol), KOH (5 mmol), CuNPs/Celite (158 mg, 5 mol % or 632 mg, 20 mol %), EG (5 mL), and DMA or CH_3_CN or H_2_O (2 mL). *T*, 130 °C, *t*, 3 h. (b) Continuous-flow condition: nitrobenzene in EG (5 mL of 0.5 M, 1 equiv) and 2 equiv of KOH; 2 mL of DMA or CH_3_CN or H_2_O; flow rate, 0.03 mL/min; and 250 mg of CuNPs/Celite 5% w/w. The reaction was monitored after 18 reactor volumes, and conversion was determined by GC-MS.

To complete the reaction optimization, the influence of polar solvents such as DMA, CH_3_CN, and water was studied. Preliminarily DMA was added to the reaction mixture, keeping constant the molar ratio of EG:nitrobenzene 35:1, and the reaction was performed under conventional batch conditions in the presence of 5 or 20 mol % of CuNPs/Celite (5% w/w). In both reactions the conversion decreased in the presence of DMA, but interestingly, when the reaction was repeated in continuous flow, we observed only an almost negligible reduction of reaction conversion to aniline (see Figure 6). The influence of DMA on nitrobenzene TH to aniline was evidenced when the EG:DMA ratio was increased to 1:1, since the reaction yield decreased from >99% to 92.5%. The same trend was also observed when CH_3_CN and H_2_O were added to the 0.5 M solution of nitrobenzene in EG: when the reaction was performed in batch, only lower than 5% of aniline was detected after 3 h of reaction, while more than 95% of aniline was present in the reaction mixture collected after 3 h of reaction in continuous flow (18 reactor volumes). We could observe that additional solvents such as DMA, acetonitrile, and water affect reaction conversion and selectivity, but the efficacy of continuous-flow technology in enhancing the reaction rate counterbalanced this detrimental effect.

The suitability of immobilized catalysts for use over several hours or even days in continuous applications is one of their most important features. We therefore placed special emphasis on monitoring the CuNPs/Celite-catalyzed reduction of nitrobenzene over a prolonged period of time, with the following selected reaction conditions being the most productive; nitrobenzene solution in EG 0.5 M, 0.03 mL/min, packed bed reactor loading of CuNPs/Celite 5% w/w, 130 °C.

**Figure 7:**
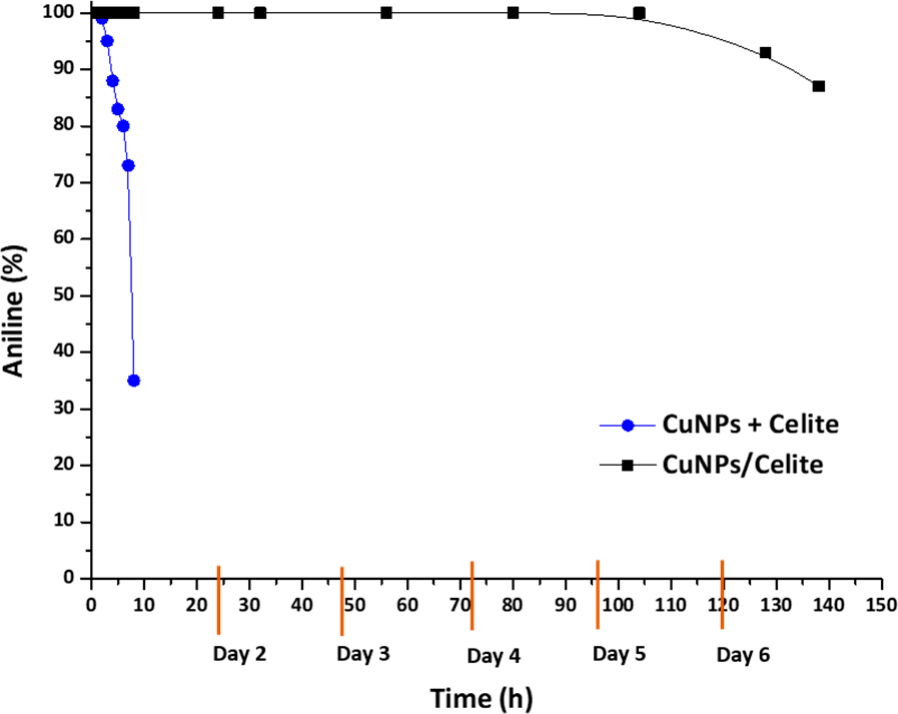
Long-run studies for the continuous reduction of nitrobenzene in the presence of CuNPs + Celite (blue curve) and CuNPs/Celite (black curve). Reaction conditions: nitrobenzene in EG (0.5 M, 1 equiv) and 2 eq KOH. Flow rate: 0.03 mL/min, 500 mg of CuNPs/Celite 5% w/w, setup B (see Figure S1B). Reaction temperature: 130 °C. Reaction yield determined by GC-MS.

Figure 7 shows that full conversion was retained for the first 2 h when the physical mixture of CuNPs + Celite was tested under continuous flow. Indeed, the reaction mixture began to show traces of unconverted starting material after 3 h, while after 4 h, the aniline yield had dropped to 88%. From this point onward (reaction times of 5 and 6 h) catalytic activity dropped dramatically and the experiment was stopped. The same experiment was repeated in the presence of CuNPs supported on Celite (CuNPs/Celite), and in this case, full conversion was maintained up to 99 h (4 days) and then remained at over 87% conversion for 145 h. A volume of 260 mL of solution was recovered in 145 h, giving a theoretical yield of 0.13 mol (12 g) of aniline; gratifyingly, 11.1 g of aniline was recovered after extraction at a yield of 93% (0.12 mol). Considering the amount of reacted nitrobenzene, the reaction was successfully performed with 0.3 mol % of catalyst, thus confirming the high efficiency of flow chemistry in increasing the reaction rate and improving catalysis performance.

The enhanced mass transfer of continuous-flow reactors combined with the high local catalyst concentration also resulted in increased reaction rates and higher turnover numbers (TONs) for the CuNPs, which increased from 20 when the reaction was performed in batch to 333 in continuous flow (Table 2). This is an extremely positive result for a non-noble metal-supported catalyst in continuous transfer hydrogenation.^68,69^ In fact, as reported in Table 2, when performed under continuous flow, this procedure granted better performance than the conventional, microwave-promoted, and microwave/ultrasound-promoted transfer hydrogenations of nitrobenzene.

**Table 2:** Comparison of Reaction Times and TONs of the CuNP-Catalyzed TH of Nitrobenzene under Conventional, Oil Bath, MW, MW/US Irradiation, and Continuous-Flow Conditions

reaction conditions	reaction time	Cu cat	TON	ref
nitrobenzene, glycerol, KOH (2 equiv), CuNPs, 130 °C, oil bath	1 h	5 mol %	20	(14)
nitrobenzene, glycerol, KOH (2 equiv), CuNPs, 130 °C, MW	20 min under MW	2.5 mol %	40	(14)
	10 min under MW/US	2.5 mol %	40	(14)
nitrobenzene (130 mmol), EG, KOH (2 equiv), CuNPs/Celite (500 mg 5% w/w), 130 °C, 0.03 uL/min flow chemistry	3.04 min	0.3 mol % 6.7474 m^2^ (Cu surface area)	333	this work

The visual inspection of the catalysts before and after the reaction showed that if fresh unsupported CuNPs are black (Figure S5, vial 1), they display a gray tint when supported on Celite (Figure S5, vial 2). However, the CuNPs/Celite catalyst showed a very light reddish color, possibly due to the formation of Cu_2_O (Figure S5, vial 3), after the long-run continuous-flow catalysis (after reaction: AR). Therefore, to better understand the results above, CuNPs/Celite 5% w/w was characterized by ICP, FTIR, DR UV–vis-NIR, and XRD with the aim of establishing structure–activity relationships.

In accordance with the estimated amount, the ICP analyses confirmed that the Cu content on the Celite surface was 50.37 ± 0.74 g/kg. ICP-OES analysis was also performed on the solution collected during flow operation at the reactor outlet to measure potential Cu leaching in 24 h. The detected copper peak was below the limit of quantification, and we could assess that its concentration was lower than 7.3 mg/kg. The quantified leaching is lower than 1.2% of solid-supported copper.

Cu particle size distribution was obtained by counting a representative number of particles by high-resolution transmission electron microscopy (HR-TEM) (see experimental part in the SI and Figure S9d). The mean NP diameter was 10 nm with a narrow size distribution mainly from 5 to 15 nm. Based on the particle size distribution, the corresponding metal specific surface area (SSA, m^2^/g) of the Cu nanoparticles (supposed to be spherical) was calculated by the equation:
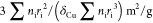
where *r*_i_ is the mean radius of the size class containing *n*_i_ particles and δ_Cu_ is the volumetric mass of Cu, equal to 8.96 g/cm^3^.

The CuNps/Celite present a SSA of 269.896 m^2^/g correspond to 17 150.8 m^2^/mol_Cu_. Considering 500 mg of CuNPs/Celite 5% w/w (25 mg of Cu as measured by ICP), the Cu surface area is 6.7474 m^2^, which corresponds to 2.36 × 10^20^ exposed Cu atoms (for detail see the SI).

The FTIR spectra of the CuNPs/Celite and the physical mixture (CuNPs + Celite) were collected and compared with the spectral features of the individual CuNPs and bare Celite (see the Figure S5). The broad band at 3433 cm^–1^ can be attributed to the O–H stretching mode of ethylene glycol and can be observed in the spectra of bare (black curve), supported (blue curve), and physically mixed (orange curve) CuNPs. Moreover, the spectrum of bare Celite (red curve) does not display this absorption band, confirming that an ethylene glycol shell formed around the NPs, with similar features being observed for glycerol.^14^ DR UV–vis-NIR characterization was carried out both on fresh, i.e., before reaction, and AR catalysts (see Figure S6). The presence of a very weak and broad absorption band at 18 200 cm^–1^, which is attributed to the surface plasmon (SPR) resonance of metallic Cu,^70^ was only observed in the fresh CuNPs/Celite sample (black line). As for the AR catalysts, bands at 43 430, 36 890, 30 980, and at 13 850 cm^–1^, which have been described in the literature as Cu^2+^ ← O^2–^ charge-transfer (CT) transitions and the d–d transitions of isolated distorted octahedral Cu^2+^ ions, respectively,^70,71^ were observed (Figure S6a, pink and green lines), meaning that CuNP oxidation takes place during the reaction, which is in agreement with the color change observed in Figures 5 and S3. Moreover, a poorly defined component was observed at 30 980 cm^–1^, and this is caused by the O^2–^ → Cu^2+^ CT transition of either dinuclear^72−74^ or trinuclear^74,75^ copper–oxygen complexes. These spectroscopic features may suggest that the AR catalyst mainly included isolated mononuclear Cu^2+^ species, i.e., that the copper NPs are partially oxidized, likely at their surface. Overall, these absorption bands are much more intense in the case of AR CuNPs/Celite (pink line) than in AR CuNPs + Celite (green line), which indicates that there is a relatively high amount of copper species on CuNPs/Celite. In addition, the band at 13 850 cm^–1^, observed for CuNPs/Celite (Figure S6b, pink line), is shifted to higher wavelengths than that for the CuNPs + Celite mixture (green line), which may suggest that a confinement effect occurs,^76^ when the CuNPs are supported on Celite rather than mechanically mixed with it.

The CuNPs alone and those supported on Celite were then investigated by using X-ray diffraction (XRD). On one hand, as shown in Figure S7, the diffraction pattern of the CuNPs (black curve) shows peaks at 43.25°, 50.43°, 74.15°, 89.91°, and 95.14°, which correspond to the (111), (200), (220), (311), and (222) planes of metallic copper in the cubic phase (file number 00–004–0836), respectively. Furthermore, the peaks at 29.93°, 36.82°, and 62.80° correspond to the (110), (111), and (220) planes of Cu_2_O in the cubic phase (file number 00–0034–1354), respectively. The relative intensities of the peaks for cubic metallic copper and Cu_2_O suggest that only a small amount of unsupported CuNPs is oxidized. On the other hand, for the supported catalyst (CuNPs/Celite) and the mechanical mixture (CuNPs + Celite), the main (111) peak for cubic Cu(0) at 43.25° (file number 00–004–0836), which was detected in the pattern of the CuNPs/Celite catalyst (red line, Figure S8), is higher in intensity than that observed for the CuNPs + Celite mixture (dark yellow line). It is worth noting that, unlike the unsupported CuNPs, no peaks for the Cu_2_O cubic phase were observed on either CuNPs/Celite or CuNPs + Celite, and this is due to (i) the lower copper amount, (ii) superimposition with the peaks of the Celite support, and possibly (iii) interactions with Celite.

Moreover, the main (111) peak of metallic copper is no longer observed in the CuNPs/Celite pattern after reaction, which means that no crystalline metallic copper was present in the AR catalyst, unlike in the fresh one (Figure 6, dark green line vs green line). Interestingly, only the peaks assigned to the support were observed, while no cubic-Cu_2_O-related peak was detected, likely meaning that the presence of this phase corresponds to a highly dispersed crystalline species.

**Figure 8:**
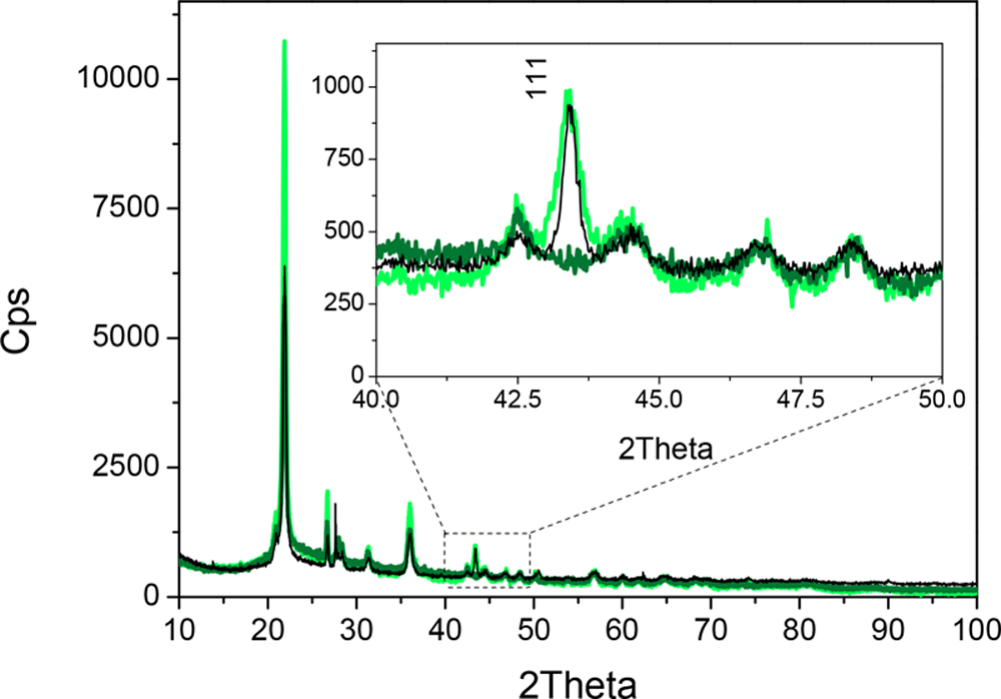
XRD patterns of the CuNPs/Celite catalyst before (green line) and after reaction (dark green line) and of the reactivated CuNPs/Celite (black line).

Being that the solvent of our system is a reducing compound, fresh ethylene glycol was then flowed through the exhausted catalyst at 130 °C in the PBR in order to reactivate/reduce the particles. Indeed, an XRD analysis of the reactivated catalyst (Figure 8, black line) showed that the intensity of the main (111) peak of metallic copper was recovered, demonstrating that (i) the Cu(0) species can be easily restored, (ii) the CuNPs/Celite catalyst is recyclable, and therefore (iii) the catalyst seems to be stable. These observations were further confirmed by the results of the SEM measurements combined with EDS analysis (reported in Figure S9) carried out on the CuNPs/Celite catalyst before (a) and after the reaction (b) and after reactivation (c).

In particular, the morphology of the catalyst is strongly modified upon reaction (Figure S9, see panel b), as the CuNP-rich regions appear brighter than the support and have a globular disk shape due to reaction mixture residue that remains despite the drying performed to prepare the sample for analysis. Nevertheless, SEM characterization showed that the original morphology of the catalyst is restored by EG flow at 130 °C, and the CuNPs are then observed once again (Figure S9, panel a vs panel c).

As the use of flow technology is challenging when compound solubility is low and based on the previous study on the influence of cosolvents, we evaluated the influence of DMF and DMA on nitronaphthalene TH.

**Figure 9:**
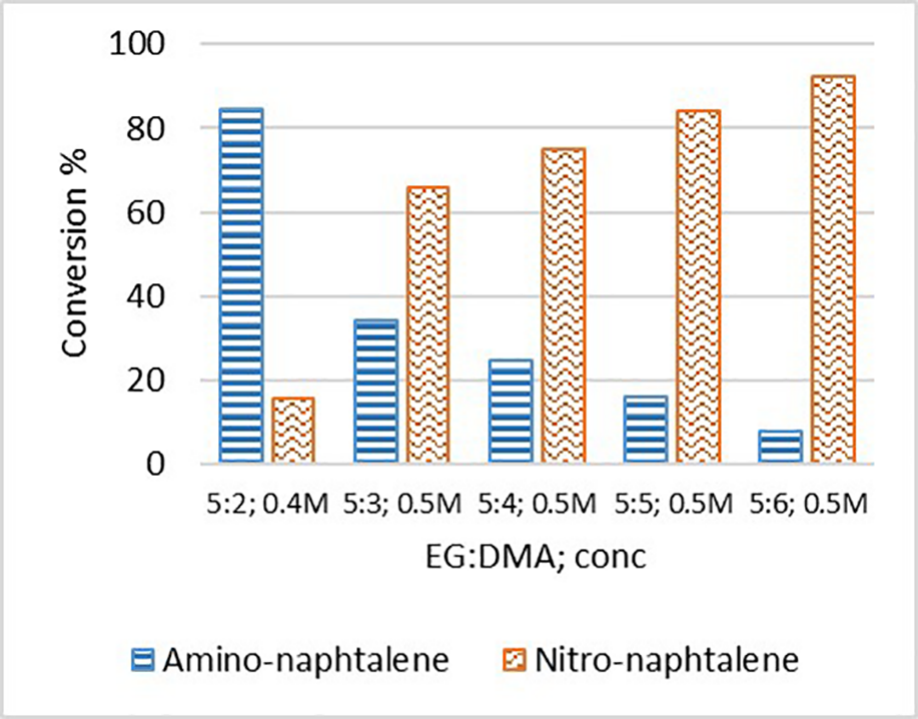
Influence of DMA as a solubilizing solvent. Tests were performed at different DMA-to-EG ratios and different nitronaphthalene concentrations. Reaction conditions: nitronaphthalene (1 equiv) in EG/DMA (concentration 0.4 or 0.5 M), 2 equiv of KOH, flow 0.03 mL/min, reaction temperature 130 °C.

**Scheme 2:**
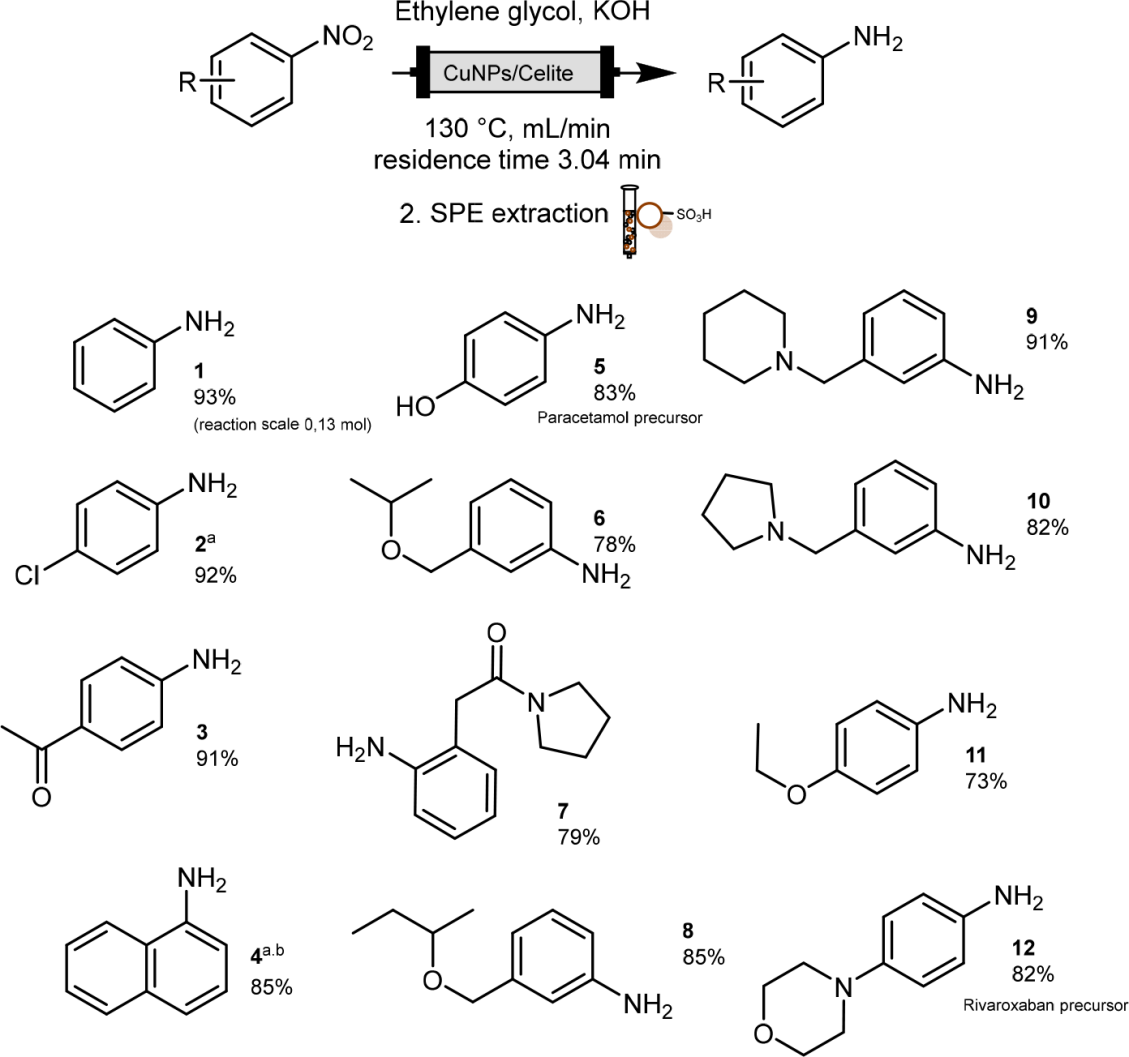
Scope of Nitrobenzene Reduction Reaction conditions: nitrobenzene derivatives (1 eq) 0.5 M in EG, 2 eq KOH, flow rate 0.03 mL/min, reaction temperature 130 °C. (a) Reaction was performed in EG/DMA 5:2. (b) Concentration of nitronaphthalene 0.4 M.

1-Nitronaphthalene crystallized and clogged tubes when dissolved at 0.5 M in EG, whereas it showed solubility at 0.05 M in EG, which made the reaction protocol, the workup, and the isolation of the desired product very difficult. As described in Figure 9, naphthalene was successfully converted to the desired amine when dissolved in a 5:2 mixture of DMA/EG. An increased amount of DMA led to reduced conversion. In order to achieve complete dissolution, the concentration was decreased to 0.4 M, and 1-aminonaphtalene was recovered in 85% yield. When 4-chloronitrobenzene (Scheme 2 product **1**.**2**) was reduced in DMF/EG, the main side product was produced via the displacement of chlorine by dimethylamine. For this reason, only DMA could be used to obtain the desired product in a 96% yield.

We next focused on expanding the scope of the reaction in order to prove its general applicability. With the aim to cover chemical diversity in terms of functional group tolerance, stability and electronic influence of not only commercially available nitrobenzene derivatives but also *ad hoc* synthesized nitroarenes were tested (cmpds 1.6–1.11, for procedures and characterization, see the SI) As shown in Scheme 2, the continuous-flow reduction of nitroarenes was studied under the most productive reaction conditions (CuNPs/Celite 5% w/w, 0.03 mL/min, 130 °C). Within the selection of 11 nitrobenzene derivatives, those substituted with amine, ether, alcohol, halogen, amido, and carbonyl functional groups were efficiently reduced to anilines with extremely high yields. As already demonstrated for the batch procedure,^14^ the reaction showed high chemoselectivity, and it was possible to reduce nitrobenzene without affecting the carbonyl functions (see product **3**). High yield was achieved in the presence of a secondary amide function, which demonstrates that continuous-flow synthesis increases the reaction rate and has a beneficial influence on the stability of acetamido derivatives toward hydrolysis and degradation (see product **7**). In order to compare the conventional and continuous synthesis of *p*-amino phenyl acetamido derivatives, the reaction was also performed in an oil bath in EG with 5 or 20 mol % Cu/Celite (5% w/w); HPLC-MS analysis was exploited to determine conversion and purity. Despite the fact that only 15% of aniline was obtained after 3 h of TH with 5 mol % catalyst and a mixture of starting material and degradation products was observed, when the amount of catalyst increased to 20 mol %, 95% conversion was reached after 1.5 h. The final purity of the *p*-amino phenyl acetamido derivative measured by HPLC was 32% (diode array at 220 nm, see Figures S10 and S11) to confirm that continuous-flow chemistry represents a more efficient and selective alternative to conventional synthesis in batch. Amine, alcohol, and ether moieties were fully tolerated by the reaction, which also proved to be efficient in the presence of ortho-substituted nitrobenzene (see Scheme 2 product **7**). Furthermore, two drug precursors were synthesized: from reduction of the nitro derivative, the *p-*amino phenol (paracetamol) and 4-morpholino aniline (Rivaroxaban) were obtained in excellent yield.

As reported in Scheme 2, all depicted anilines were isolated in high yields, and the desired products were obtained by either solid phase or liquid–liquid extraction. The high polarity of EG imposed a limit on liquid–liquid extraction as large volumes of organic solvents were required. On the other hand, pure aniline derivatives were isolated using ion-exchange resins (IXR); the crude was treated with DOWEX sulfonic resin, and the captured anilinium salts were either purified from trace starting materials or from side products by washing the resin with methanol and DCM. Pure free anilines were released in the presence of ammonia in methanol solution and dried under vacuum; all products were analyzed without any further purification.

The reaction was scaled up by means of larger bad pack reactors that were filled with 1.1, 2.5, and 5 g of catalyst. The reactor volume and the void volumes were measured as reported in the SI Section 1.2 (Packed Bed Reactors), and reactions were performed varying the flow that was gradually increased. Conversion and yield were measured by means of GC-MS analysis, and results are shown in Table 3. We evidenced that a residence time of approximately 7 min could provide full conversion and selectivity for all the reactors, while when the residence time decreased to 4 min almost complete conversion was achieved in all the cases. In fact, when the flow was increased to 1 mL/min, aniline was obtained in 93.8% yield when 5 g of catalyst were employed. After 24 h of continuous operation, 720 mmol of nitrobenzene were converted with a productivity of 29.4 mmol/h (2,738 g/h).

**Table 3:** Scaling Up of Cu-Catalyzed Nitrobenzene Reduction in EG under Continuous Flow

en.^a^	CuNPs/Celite (w/w %)	flow rate (mL/min)	resid. time (min)	aniline^b^	nitrobenzene^b^	azobenzene^b^	azoxybenzene^b^
1	1.1 g (5% w/w)	0.132	7.2	>99	-	-	-
		0.22	4.3	98.1	1.3	-	0.6
		0.308	3.1	66.6	32.7	-	0.7
		0.440	2.2	52.9	45.7	-	1.4
2	2.5 g (5% w/w)	0.3	6.8	>99	-	-	-
		0.5	4.1	>99	-	-	-
		0.75	2.7	73	24.7	-	2.3
		1.0	2.0	39.6	51.9	1.5	6.8
3	5 g (5% w/w)	0.600	7.0	>99	-	-	-
		1.0	4.2	98.3 (97%)^c^	1	-	0.7
		1.4	3.0	67	30.2	-	2.8

a Reaction performed using a 0.5 M solution of nitrobenzene in EG, KOH 2 eq. Reaction temperature: 130 °C.

b Reaction yield determined by GC-MS.

c Reaction was performed in the presence of reused EG.

Due to the fact that organic solvents are often discarded after a single use and can account for up to 90% of the process by mass, we attempted to purify and reuse the solvent.^77,78^ After capturing of the desired aniline by means of the IXR, the recovered EG was distilled and reused in the reduction of the nitrobenzene; EG of recovered and purified EG was preliminarily checked by NMR (see the experimental procedure). The reaction performed in the presence of distilled EG showed 97% yield to demonstrate that KOH represents the waste of the reaction.

**Figure 10:**
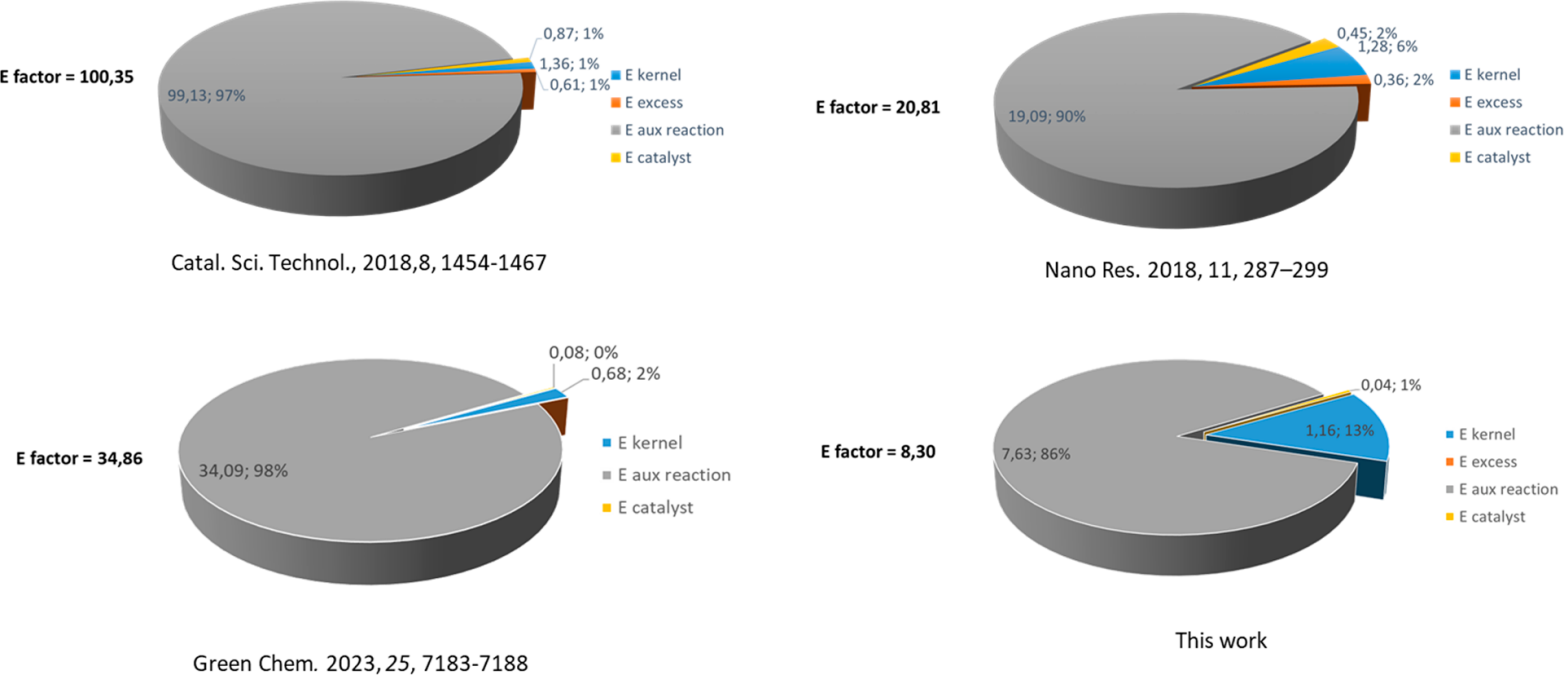
E-factor distribution analysis.

Different aspects influence the practical feasibility and the cost of a process such as waste amount, safety, operational simplicity, and availability of necessary equipment. The greenness and sustainability of our procedure was compared with three representative examples of continuous nitrobenzene transfer hydrogenation since, with respect to hydrogenation, they do not require specifically designed equipment for producing and/or handling gaseous H_2_ at high pressure (see Figure 10). By means of the Cu-catalyzed continuous procedure, aniline was obtained with a mass-dependent metric (E-factor) of 8.30. In order to evaluate the sustainability improvement of our protocol, the E-factor was also calculated for the whole procedure (20.19) and the detailed calculation is shown in the SI. Other examples of continuous nitrobenzene TH with Cu catalysts in alcohol have not yet been described, therefore we selected three protocols based on Pd/NaBH_4_,^42^ Bi–Fe catalyst/hydrazine,^47^ or Pd/NH_3_·BH_3_.^52^ All published procedures are not focused on green chemistry metrics, therefore the work up and purification could not be included in the calculation (see the SI and Figure 10). Due to the EG recovery and reuse, as well as the IXR regeneration, the E-factor value associated with our flow setup is lower with respect to other alternatives.

## Conclusions

A simple and readily upscalable catalytic system for the transfer hydrogenation of nitrobenzene has been reported herein. The reaction is performed in the presence of a robust CuNPs-supported catalyst and ethylene glycol, which acts as both the sacrificial hydrogen donor and the solvent. The CuNPs/Celite catalyst led to full conversion being achieved for up to 99 h (4 days), and conversion then remained at over 87% for 170 h (7 days), highlighting the stability of the system. Moreover, the TON increased from 20 when the reaction was performed in batch mode to 333 under a continuous flow. Although DR UV–vis-NIR characterization demonstrated the presence of oxidized copper after reaction, the CuNPs/Celite catalyst can be easily reactivated, as shown by XRD analysis and EDS measurements combined, and then reused. Compared to conventionally heated and MW- and MW/US-promoted protocols, continuous-flow chemistry increases the efficiency of the catalytic process, increasing the reaction rate and productivity. Moreover, despite the principle limitation of the low solubility of several nitrobenzenes, the reaction can be performed in a mixture of EG and either DMA or DMF, providing excellent results.

## Supporting Information Available

Additional experimental details for continuous-flow synthesis and catalyst characterization, experimental procedure for intermediates synthesis, E-factor analysis, and NMR spectra for all compounds (PDF)

## ABBREVIATIONS

API: active pharmaceutical ingredient
AR: after reaction
DMA: dimethylacetamide
DMF: dimethylformamide
EG: ethylene glycol
GC: gas chromatography
ICP: inductively coupled plasma
MW: microwave irradiation
MW/US: combined microwave and ultrasound irradiation
NP: nanoparticle
PBR: packed bed reactor
SEM: scanning electron microscope
TH: transfer hydrogenation
TLC: thin layer chromatography
XRD: X-ray powder diffraction
