# Supplementary material for: Copper-Catalyzed Continuous-Flow Transfer Hydrogenation of Nitroarenes to Anilines: A Scalable and Reliable Protocol
Source: Org Process Res Dev. 2023 Dec 21;28(5):1515–28. doi: 10.1021/acs.oprd.3c00144 (PMC11110069; doi:10.1021/acs.oprd.3c00144)
Supplement: Supplementary file 1 — op3c00144_si_001.pdf [file op3c00144_si_001.pdf]

Supporting information

Copper-catalyzed continuous-flow transfer hydrogenation of nitroarenes to anilines: a scalable and reliable protocol

Katia Martina, †*[a] *Maria Jesus Moran,* †[a] *Maela Manzoli,*[a] *Mikhail Trukhan,*[a] *Simon Kuhn,* [b] *Tom Van Gerven,* [b] *Giancarlo Cravotto*[a]

AUTHOR ADDRESS

[a] Dipartimento di Scienza e Tecnologia del Farmaco and NIS Interdepartemental Centre for Nanomaterials for Industry and Sustainability, University of Turin, via Pietro Giuria 9, 10125 Turin (Italy). *Email: katia.martina@unito.it

[b] Department of Chemical Engineering, KU Leuven, Celestijnenlaan 200F, 3001 Leuven, Belgium

†These authors contributed equally to this work

Table of Contents

1. General Materials and Methods S3

1.2 Set-up of the continuous flow system S4

1.2 Packed bed reactors S5

2. General procedure for catalyst synthesis (CuNPs/Celite 5 w/w %) S7

3. Procedure for the synthesis of intermediates S7

4. Continuous nitro benzenes reduction: products characterization S11

5. Catalyst characterization S15

5.1 Specific Surface Area and of number of exposed Cu site on the catalyst surface. S19

6. Batch synthesis of 2-(2-aminophenyl)-1-(pyrrolidin-1-yl)ethan-1-one (7): stability and purity assessment S20

7. E-factor Analysis S21

8. NMR Spectra of intermediate and final compounds S25

9. References S36

1. General Materials and Methods

All commercially available reagents and solvents were used without further purification. The used ethylene glycol contained no less than 99 wt.% of the main substance. Reactions were monitored by TLC on Merck 60 F254 (0.25 mm) plates (Milan, Italy), which were visualized by UV inspection and/or by heating after a spraying with 0,5% ninhydrin in ethanol. NMR spectra (Jeol ECZ-R 600 MHz and 125 MHz for ^{1}H and ^{13}C, respectively) were recorded. Chemical shifts were calibrated to the residual proton and carbon resonances of the solvent, $CDCl_3$ (δH = 7.26, δC = 77.16). Chemical shifts (δ) are given in ppm, and coupling constants (J) in Hz. GC conditions were: injection split 1:10, injector temperature 250 °C, detector temperature 280 °C. Gas carrier: helium (1.2 mL/min), temperature program: from 50 °C (5 min) to 100 °C (1 min) at 10 °C/min, to 230 °C (1 min) at 20 °C/min, to 300 °C (5 min) at 20 °C/min. The cations were determined with a Perkin Elmer Optima 7000 (Perkin Elmer, Norwalk, Connecticut, USA) inductively coupled plasma-optical emission spectrometer (ICP-OES).

DR UV–Vis-NIR spectra were run on a Varian Cary 5000 spectrophotometer, working in the 190-2500 nm range of wavenumbers. The spectra are reported in accordance with the Kubelka-Munk function: [$f(R_\infty)=(1-R_\infty)^2/2R_\infty$; R_∞= reflectance of an “infinitely thick” layer of the sample].

All FT-IR spectra were acquired with a Bruker Equinox 55 spectrometer equipped with a MCT detector at a resolution of 4 cm^{-1}, averaging 64 scans.

X-Ray Diffraction (XRD) patterns were collected with a PW3050/60 X'Pert PRO MPD diffractometer from PANalytical working in Bragg–Brentano geometry, using as a source the high-powered ceramic tube PW3373/10 LFF with a Cu anode (using Cu Kα1 radiation λ = 1.5406 Å) equipped with a Ni filter to attenuate Kβ. Scattered photons were collected by a real time multiple strip (RTMS) X'celerator detector. Data were collected in the $10° \leq 2\theta \leq 100°$ angular range, with 0.02° 2θ

steps. The powdered samples were examined in their as-received form and posed in a spinning sample holder in order to minimize preferred orientations of crystallites.

The morphology and composition of the fresh CuNPS/Celite, and those after reaction and reactivation, were investigated by scanning electron microscopy (SEM; Zeiss Evo50) operating at 20 kV using an energy-dispersive X-ray detector (EDS).

Cu NPs size was measured by high resolution transmission electron microscopy (HR-TEM) analysis; it was carried out by using a 300 kV JEOL 3010-UHR microscope equipped with a LaB6 filament and with X-ray EDS analysis by a Link ISIS 200 detector. Digital micrographs were acquired by a (2k x 2k)-pixel Ultrascan 1000 CCD camera and were processed by Gatan digital micrograph.

1.2 Set-up of the continuous flow system

Flow chemistry was carried out in two different types of reactors:

A. a syringe pump (Harvard Apparatus), a packed bed reactor (PBR), a heating mantle and an oil bath (see Figure S1-A). All units were connected using PTFE tubing (o.d. 1/16 in., i.d. 0.75 mm) and standard HPLC fittings (all with an i.d. of 0.70 mm). Two filters were display at both sides of the packed bed reactor unit (porous size, dimension). The PBR was heated by immersing the reactor inside the oil bath (130°C).

B. a Syrris Asia flow Chemistry System equipped with a Glass Column Reactor to host the packed bed reactor (PBR) (see Figure S1-B). This Solid Phase Reactor allow the use of solid-supported catalyst at a temperature up to 150°C. The reactor is commercially available, made of glass and PTFE 100 mm long offering a wide range of volumes. In the present study the the 5.6 mL and the 12 mL reactor with Adjustable End have been employed. The columns can be heated by mounting on the Asia Heater using the Solid Phase Adaptor. The reactor was located in an Asia Heater module. All units were connected using PTFE tubing (o.d. 1/8 in., i.d. 1/16 inch). The PBR was heated at 130°C.

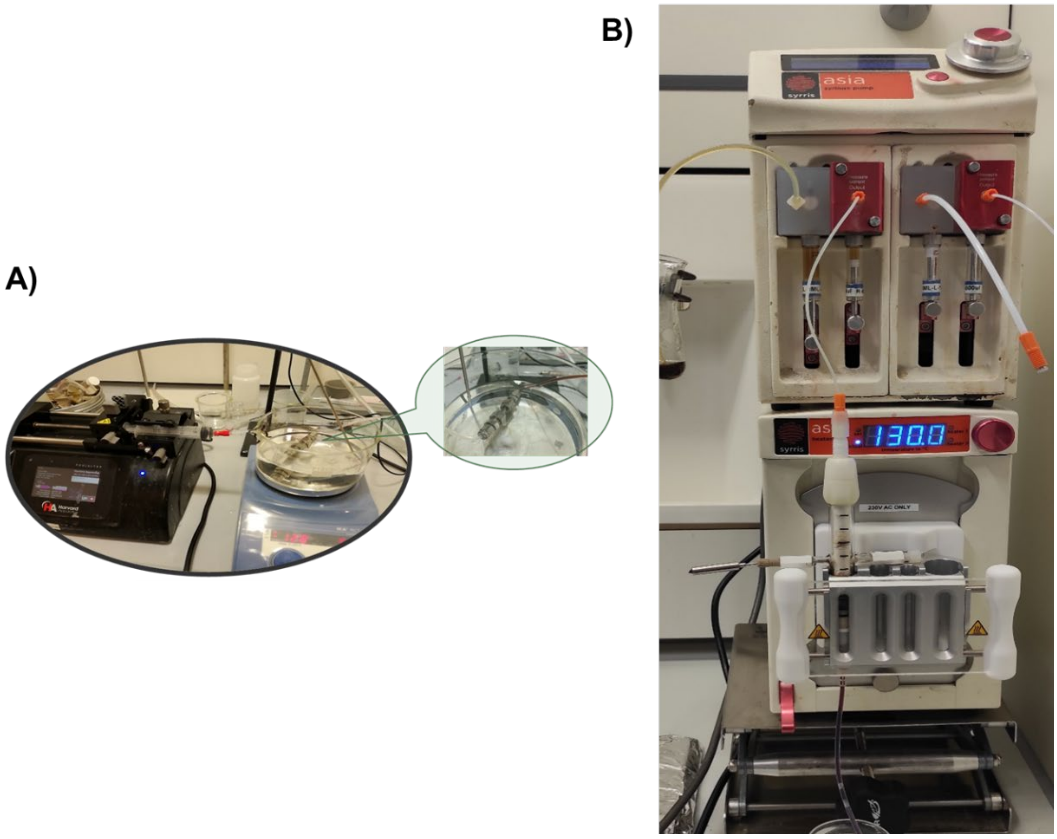

Figure S1: Representation of both the continuous flow systems: (A) setup reactor A (B) setup reactor B

1.2 Packed bed reactors

In both cases, the total volume (V_t) of the packed bed reactor and the internal void volume once packed with the catalyst (R_v) were determined:

A. The packed bed reactor has a length of 2,6 cm and a i.d. of 0,4 cm. The total volume of the reactor was determined:

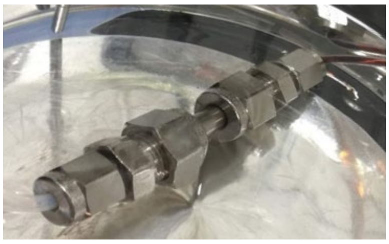

Figure S2: Detail of continuous flow systems PBR A

$$\mathbf{V_t}: (\pi\cdot r^2)\cdot l=[3,14\cdot(0.2cm)^2]\cdot 2,6cm: \mathbf{0,327\ cm^3}$$

Once the packed bed reactor was filled with the catalyst, the internal volume was determined by weighing the freshly PBR (Mass 1) and then pumping a solvent (ethanol) through the reactor to fill the void with it. The packed bed reactor was then weighed again (Mass 2). The void volume was determined by V_0 = (Mass 2 – Mass 1)/ solvent density.

V_0 = (Mass2 – Mass1)/ethanol density: **0,149 cm³**

B. The packed bed reactor has a length of 0,8 cm and a i.d. of 1 cm. Only a small portion of the reactor (0.8 cm) were filled. The used volume was determined:

$$\mathbf{V_t}: (\pi \cdot r^2) \cdot l = [3{,}14 \cdot (0{,}5\ cm)^2] \cdot 0{,}8\ cm = \mathbf{0.628\ cm^3}$$

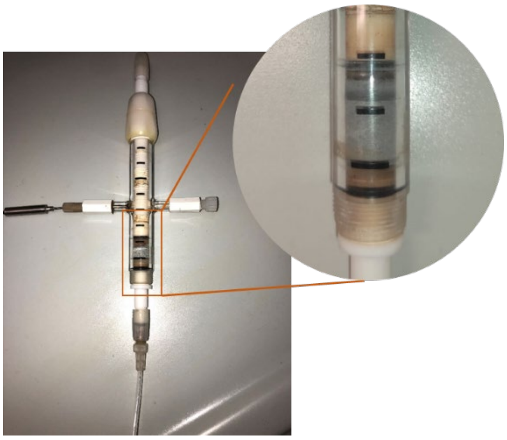

Figure S3: Detail of continuous flow systems PBR B

The internal volume was determined by weighing the freshly PBR (Mass 1) and then pumping a solvent (ethanol) through the reactor to fill the void with it. The packed bed reactor was then weighed again (Mass 2). The reactor volume was determined by Rv = (Mass 2 – Mass 1)/ solvent density.

$\mathbf{V_0}$ = (Mass2 – Mass1)/ethanol density: **0,292 cm³**

The measurements were repeated with the Asya Sirrys reactors filled with

1.1 gram of catalyst reactor diameter 0,8 cm length 6,9 cm; $\mathbf{V_t}$ = 3,412 cm^3 and $\mathbf{V_0}$ = 0,955 $cm^3$2,5 gram of catalyst reactor diameter 1,6 cm length 3,5 cm; $\mathbf{V_t}$ = 7,336 cm^3 and $\mathbf{V_0}$ = 2,04 cm^3

and 5,0 g of catalyst diameter 1,6 cm length 7 cm $\mathbf{V_t}$ = 14,672 cm^3, $\mathbf{V_0}$ = 4,2 cm^3

2. General procedure for catalyst synthesis (CuNPs/Celite 5 w/w %)

306 mg of copper (II) sulfate ($CuSO_4 \cdot 5H_2O$) were dissolved in 48 mL of H_2O/ethylene glycol (5:1) (0,025 M) was stirred and was followed dropwise by addition of 2 M NaOH aqueous solution to adjust the solution pH up to 11. The supported material (Celite® 545, 1.5 g) was then added. After stirring for 10 min, 0.5 M $NaBH_4$ in water was added into the flask while sonicating in the ultrasonic bath in order to have well dispersed particles. The deep blue solution gradually became colourless, and then it turned burgundy, which shows the formation of copper colloid. The copper nanoparticles supported on celite (CuNPs/Celite) were filtered on a Büchner funnel with a sintered glass disc with water and methanol being used to wash the catalyst.

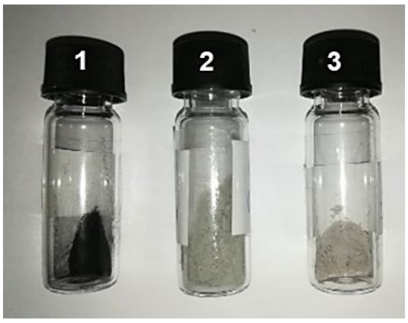

Figure S4: CuNPs/Celite supported catalysts before and after continuous flow reaction (1) CuNPs, (2) CuNPs/Celite, (3) CuNPs/Celite After Reaction (AR)

3. Procedure for the synthesis of intermediates

1-(isopropoxymethyl)-3-nitrobenzene (1.6)

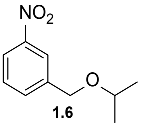

A solution of m-nitro benzyl bromide (700 mg, 3,2 mmol) in 15 mL of iPrONa/isopropanol (0.1M) was heated to 50°C under magnetic stirring o.n. After partial removal of the solvent, water and EtOAc were added and the final product extracted, AcOEt was washed three times, Na_2SO_4 anhydrous was added to the organic phase and filtered to obtain the desired product in 67% yield.

^1H NMR (600 MHz, $CDCl_3$) δ 8.19 (s, 1H), 8,09 (d, J =9 Hz, 1H),7.66 (d, J = 9 Hz, 1H), 7.48 (t, J = 9 Hz, 1H) 4.57 (s, 2H), 3,72-3,68 (m, 1H), 1.23 (d, J= 7 Hz, 6H), ppm;

NMR in agreement with literature data.[1]

2-(2-nitrophenyl)-1-(pyrrolidin-1-yl)ethan-1-one (1.7)

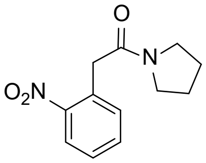

1.7

A solution of o-nitro phenyl acetic acid (700 mg, 3,86 mmol) and 1 mL of thionyl chloride (13,8 mmol) in 5 mL of toluene was heated to reflux for 20 min. Solvent was removed under vacuum, and 20 mL chloroform were added to dissolution, and a solution of pyrrolidine (5.79 mmol) and 300 uL of pyridine in 5 mL of chlorofom were added dropwise at 0°C. the reaction was left under magnetic stirring 2 hr. After removal of the solvent, water and EtOAc were added and the final product extracted, AcOEt was washed three times, Na2SO4 anhydrous was added to the organic phase and filtered. The crude was dried under vacuum and the desired product obtained by column chromatography separation (PE:EcOAt 7:3) in 47% yield.

^1H NMR (600 MHz, $CDCl_3$) 8.03 (dd, J = 9Hz, 0.9 Hz, 1H), 7.59 (td, H=7 Hz, 0.9 Hz, 1H), 7.4 (td, J= 8Hz, 1Hz, 1H), 7.33 (d, J = 8 Hz, 1H), 3,96 (s, 2H), 3,53 (t, J = 7 Hz, 2H), 3,45 (t, J = 7Hz, 2H), 1.98 (q, J = 7 Hz, 2H), 1.85 (q, J = 7 Hz, 2H)

NMR in agreement with literature data.[2]

1-(sec-butoxymethyl)-3-nitrobenzene (1.8)

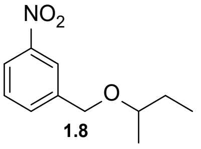

1.8

1-(bromomethyl)-3-nitrobenzene (400 mg,1.8 mmol) was dissolved in 2 ml of isobutyl alcohol. 3 equivalents of potassium hydroxide (311 mg, 5.5 mmol) were added to the solution. The resulting mixture was stirred for 8 hours at room temperature. Upon completion of the reaction, the solvent was removed, the mixture was poured into 10 ml of water and extracted with ethyl acetate twice (5x2). The organic layer was dried over sodium sulfate and evaporated on a rotary evaporator. The product was purified by column chromatography using petroleum ether-ethyl acetate 7:3 to give 343 mg of a pale yellow oil. Yield was 88%.

^{1}H NMR (600 MHz, DMSO-d^6) 8.12 (s, 1H), 8.09 (d, J = 7Hz, 1H), 7.73 (d, J = 7Hz, 1H), 7.61 (t, J = 7Hz, 1H), 4.61 d (d, J = 7Hz, 1H), 4.52 (d, J = 7Hz, 1H), 3-42-3,4 (m, 1H), 1.54-1.38 (m, 2H), 1.10 (d, J = 7H, 3H), 0,84 (t, J=7Hz, 3H)

1-(3-nitrobenzyl)piperidine (1.9)

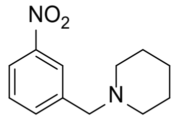

1.9

To a solution of m-nitro benzyl bromide (700 mg, 3,2 mmol) in 23,5 mL of acetonitrile, 479 uL of piperidine (4.86 mmol) were slowly added, the reaction was left under magnetic stirring 3 hr. After partial removal of the solvent, water and EtOAc were added and the final product extracted, AcOEt was washed three times, Na2SO4 anhydrous was added to the organic phase and filtered to obtain the desired product in 75% yield.

^{1}H NMR (600 MHz, $CDCl_3$) δ 8.16 (s, 1H), 8,06 (d, *J* =9 Hz, 1H), 7.65 (d, *J* = 9 Hz, 1H), 7.44 (t, J = 9 Hz, 1H) 3,51 (s, 2H), 2,35 (m, 4H), 1.55 (m, 4H), 1.41 (m, 2H) ppm;

NMR in agreement with literature data. [3]

1-(3-nitrobenzyl)pyrrolidine (1.10)

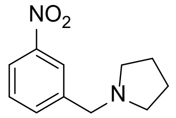

1.10

To a solution of m-nitro benzyl bromide (700 mg, 3,2 mmol) in 23,5 mL of acetonitrile, 405 uL of pyrrolidine (4.86 mmol) were slowly added, the reaction was left under magnetic stirring 3 hr. After partial removal of the solvent, water and EtOAc were added and the final product extracted, AcOEt was washed three times, Na_2SO_4 anhydrous was added to the organic phase and filtered to obtain the desired product in 87% yield.

^1H NMR (600 MHz, $CDCl_3$) δ 8.15 (s, 1H), 8,05 (d, J =9 Hz, 1H), 7.64 (d, J = 9 Hz, 1H), 7.43 (t, J = 9 Hz, 1H) 3.6 (s, 2H), 2,48 (m, 4H), 1.75 (m, 4H) ppm;

NMR in agreement with literature data.[3]

1-ethoxy-4-nitrobenzene (1.11)

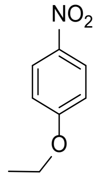

1.11

The product was obtained following a slightly modified literature procedure.[4]To a solution of 4- nitro phenol (1mmol) in dioxane , K_2CO_3 (2 mmol) and ethyl iodine (2 mmol) were added the reactrion was left under reflux 5 hours. The crude was dried under vacuum and ethyl acetate and water were added. The organic layer was washed three times and the final product isolated in pure form.

1H NMR (600 MHz, $CDCl_3$) δ 8.18–8.17 (d, 2H), 6.93–6.91 (d, 2H), 4.13 (q, 2H), 1.45 (t, 3H)

NMR in agreement with literature data[4]

4-nitrophenyl morpholine (1.12)

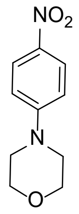

1.12

The product was obtained under solvent free condition with a slightly modified procedure with respect to the literature. 4-nitrochlorobenzene (0.5 g, 3,2 mmol), morpholine (835 mg, 9.6 mmol), and sodium carbonate (169 mg, 1.6 mmol) in a 5 mL round-bottom flask was stirred at 100 °C for 7 h. The crude was dried under vacuum and water was added to precipitate the desired product. The solid was collected by filtration, rinsed with water and dried to afford 4-nitrophenyl morpholine (**1.12**). Yellow solid (isolated yield: 97%); ^{1}H NMR (600 MHz, CDCl3) δ 8.14–8.12 (d, 2H), 6.83–6.82 (d, 2H), 3.85 (t, 4H), 3.36 (t, 4H)

NMR in agreement with literature data[5]

4. Continuous nitro benzenes reduction: products characterization

Products were analyzed using ^{1}H NMR and ^{13}C NMR spectroscopy and GC-MS chromatography.

Aniline (1):

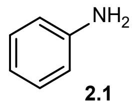

2.1

^{1}H NMR (600 MHz, $CDCl_3$) δ 7.16 (t, J = 6 Hz, 2H), 6.76 (t, J = 9 Hz, 1H), 6.69 (t, J = 6 Hz, 2H), 3,62 (s, 2H) ppm;

Isolated yield 93% (reaction scale 0.13 mol).

These data are in agreement with literature data. [7]

p-Chloroaniline (2):

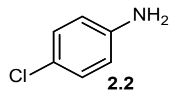

2.2

^{1}H NMR (600 MHz, DMSO-d[6]) δ 6.97 (d, J = 9 Hz, 2H), 6.51 (d, J = 9 Hz, 2H) 5.2 (s, 2H) ppm;

Isolated yield 92%.

These data are in agreement with literature data. [7]

***p*-Aminoacetophenone (3):**

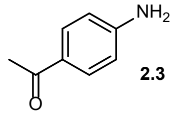

2.3

^{1}H NMR (600 MHz, DMSO-d6) δ 7.62 (d, J = 9 Hz, 2H), 6.52 (d, J = 9 Hz, 2H), 5,99 (s, 2H), 2,33 (s, 3H) ppm;

Isolated yield: 91%.

These data are in agreement with literature data. [8]

1-Naphthylamine (4):

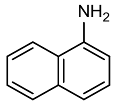

2.4

^{1}H NMR (600 MHz, $CDCl_3$) δ 7.83 (ddd, 2H), 7.46 – 7.45 (m, 2H), 7.33-7.27 (dt, 2H), 6.80 (dd, 1H), 4.25 (br.s, 2H) ppm.

Isolated yield: 85%.

These data are in agreement with literature data. [8]

***p*-Aminophenol (5):**

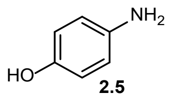

2.5

^{1}H NMR (600 MHz, DMSO-d6) δ 8.3 (s, 1H) 6.42 (d, J = 9 Hz, 2H), 6.38 (d, J = 9 Hz, 2H), 4,32 (s, 2H) ppm;

Isolated yield: 83%

These data are in agreement with literature data. [8]

3-(isopropoxymethyl)aniline (6)

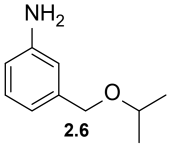

2.6

^{1}H NMR (600 MHz, $CDCl_3$) δ 7.10 (t, J = 6Hz, 1H), 6.72-6.69 (m, 2H), 6.58 (d, J = 7 Hz, 1H), 4.42 (s, 2H), 3.67-3.64 (m, 3H), 1.20 (d, J= 7 Hz, 3H) ppm;

^{13}C NMR (150 MHz, $CDCl_3$), 146.5, 140.4, 129.3,177.9,114.28, 114.26, 70.9, 70.05, 22,19 ppm

Isolated yield 78%.

2-(2-aminophenyl)-1-(pyrrolidin-1-yl)ethan-1-one (7)

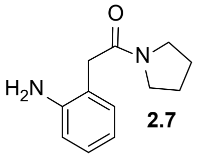

2.7

^{1}H NMR (600 MHz, DMSO-d6) 7.0 (m, 2H), 6.77 (d, J= 9Hz), 1H), 6.64 (t, H=9 Hz, 1H), 3.5 (s, 2H overlaps solvent), 3.46 (t, 2H, overlaps NH2), 3,33 (t, 2H, overlaps NH2), 1.82 (m, 2H), 1.72 (m, 2H) ppm;

^{13}C NMR (150 MHz, DMSO-d6) 169.3, 144.7131.06, 128.0, 121.8, 119.17,117.3, 46.9, 46.1, 38,6, 26,16, 24,45

Isolated yield 79%.

3-(*sec*-butoxymethyl)aniline (8)

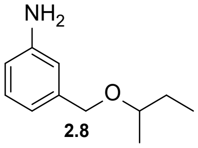

2.8

^{1}H NMR (600 MHz, DMSO-d6) δ 6.94 (d, J =8 Hz, 1H), 6.53 (s, 1H), 6.44-6.42, 3.75 (m, 2H), 5,0 (s, 2H), 4,3 (d, J =12 Hz, 1H), 4.29 (d, J =12 Hz, 1H), 3.34 (m, 1H, overlapped to DMSO),1.5-1.4 (m, 2H), 1,09 (d, J =7 Hz, 3H), 0,85 (t, J =7 Hz, 3H) ppm;

^{13}C (150 MHz, $CDCl_3$) 146.5, 140.5, 129.3, 117.9, 114.28, 114.26, 70.9, 70.1, 31.0, 22,1 ppm

Isolated yield 85%.

3-(piperidin-1-ylmethyl)aniline (9)

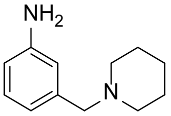

2.9

^{1}H NMR (600 MHz, $CDCl_3$) δ 7.08 (t, J = 7 Hz, 1H), 6.68 (m, 2H), 6.59-6.56 (d, J= 7Hz, 1H), 3,62 (brs, 2H), 3.37 (s, 2H), 2,38 (m, 4H) 1.57-1.54 (m, 4H), 1.43 (m, 2H) ppm; Isolated yield 91%.

These data are in agreement with literature data.[9]

3-(pyrrolidin-1-ylmethyl)aniline (10)

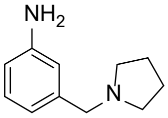

2.10

1H NMR (300 MHz, DMSO-d6) δ 6.88 (t, J = 7 Hz, 1H,), 6.49 (s, 1H),6.32-6.35 (m, 2H), 4.9 (s, 2H) 2,34 (m, 4H), 1.61 (m, 4H) ppm;

Isolated yield 82%.

NMR in agreement with literature data.[3]

4-etoxy aniline (11)

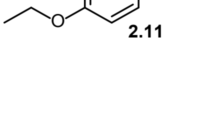

2.11

^{1}H NMR (300 MHz, DMSO-d6) δ 6.74 (d, J = 9 Hz, 2H), 6.64 (d, J = 9 Hz, 2H), 3,96 (q, J = 7Hz, 2H), 3,40 (brs, NH2), 1.36 (t; J = 7 Hz, 3H) ppm;

Isolated yield 73%

NMR in agreement with literature data[10]

4-morpholino aniline (12)

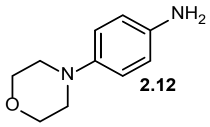

^{1}H NMR (300 MHz, $CDCl_3$) δ 6.79 (d, J = 9 Hz, 2H), 6.66 (d, J = 9 Hz, 2H), 3,84 (d, 2H), 3,01 (d; 2H) ppm;

Isolated yield 82%

NMR are in agreement with literature data[11]

5. Catalyst characterization

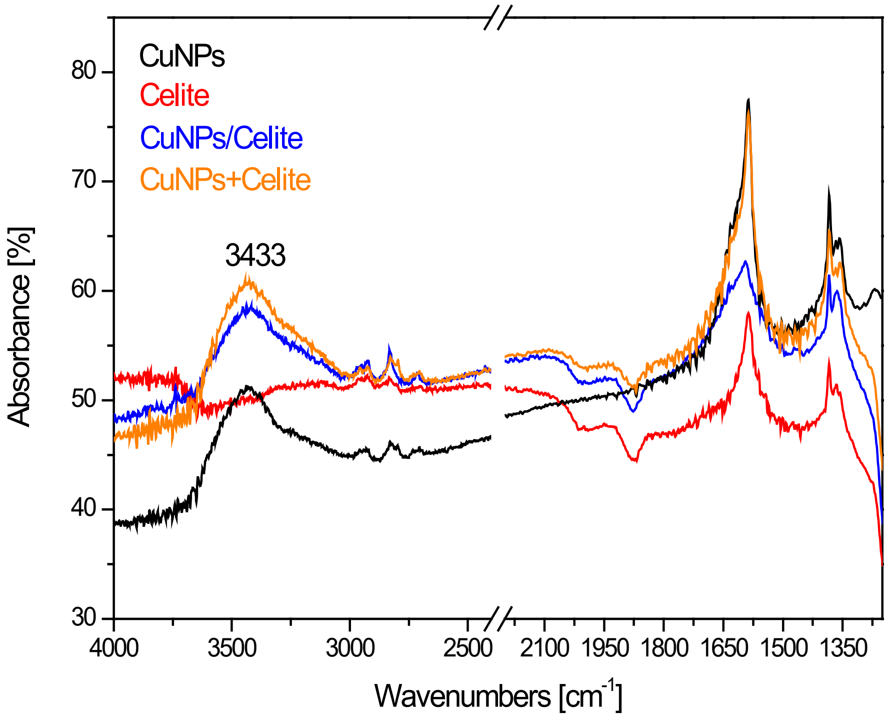

Figure S5: FTIR spectra of CuNPs (black line), Celite (red line), CuNPs/Celite (blue line) and CuNPs+Celite (orange line).

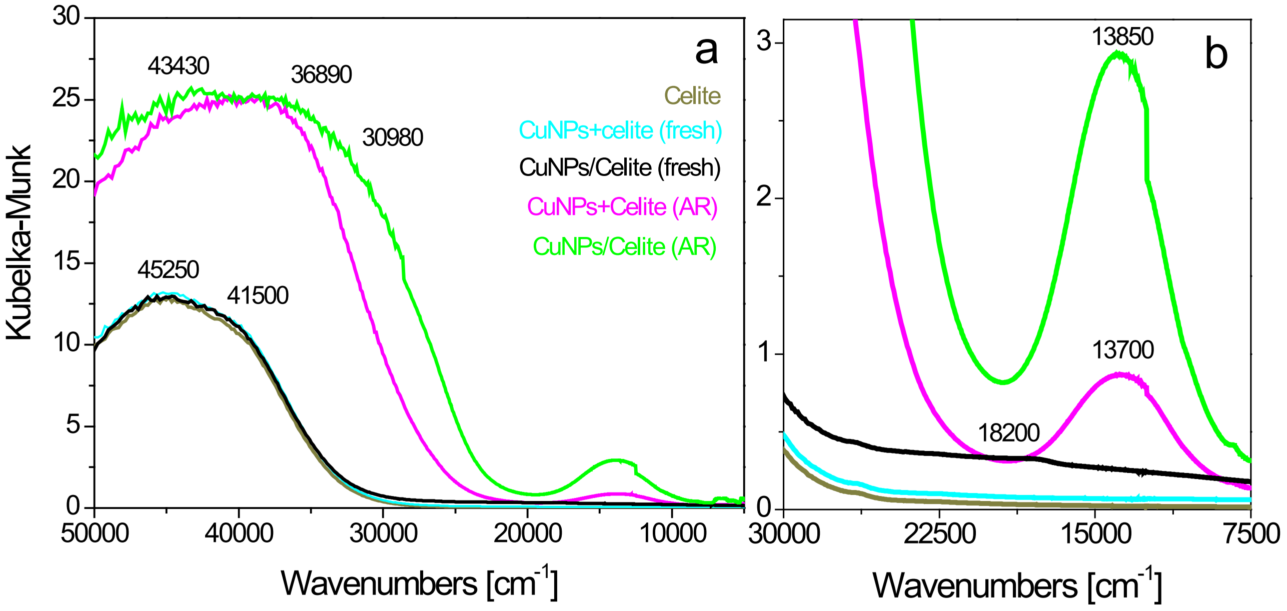

Figure S6: (a) DR UV-Vis-NIR spectra of Celite (brown line), CuNPs+Celite (fresh, cyan line), CuNPs/Celite (fresh, black line), CuNPs+Celite (AR) (pink line) and CuNPs/Celite (AR) (green line). (b) zoom of the 30000-13850 cm^{-1} spectroscopic range.

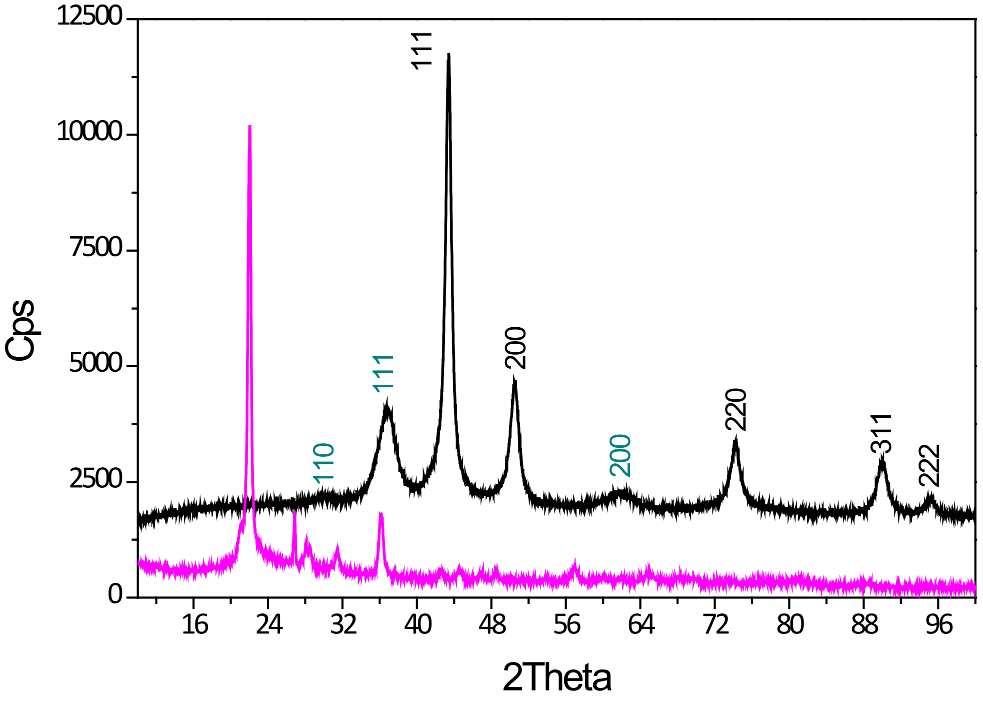

Figure S7.: X-ray powder diffraction patterns of CuNPs (black line) and Celite (pink line).

The XRD pattern of the celite support has been reported for the sake of comparison. Commercial celite is a mixture of different crystalline phases that we tentatively assigned to aluminium potassium

silicate in the hexagonal phase (most intense peak at 22°, file number 00-002-0297), and likely cubic CaO (file number 00-001-1160), cubic Na2O (00-003-1074) and tetragonal KO2 (00-002-0460).

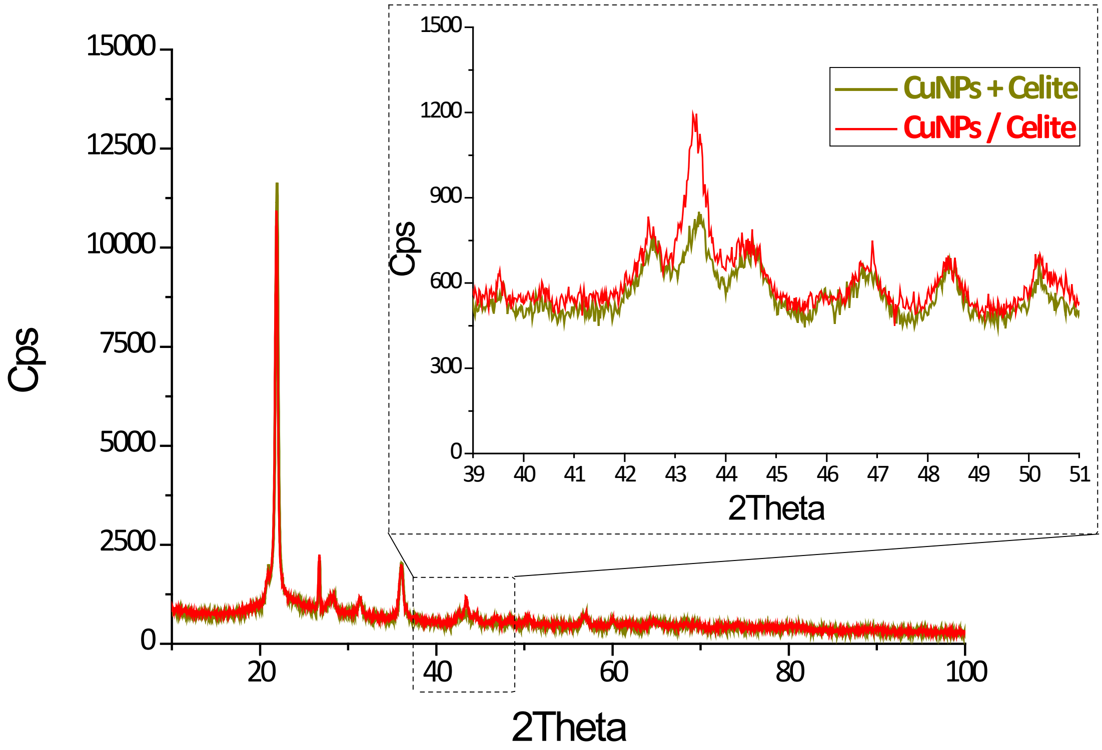

Figure S8: X-ray powder diffraction patterns of CuNPs/Celite (red line) and CuNPs + Celite (dark yellow line). Inset: zoom in the 39°-51° range of 2Theta.

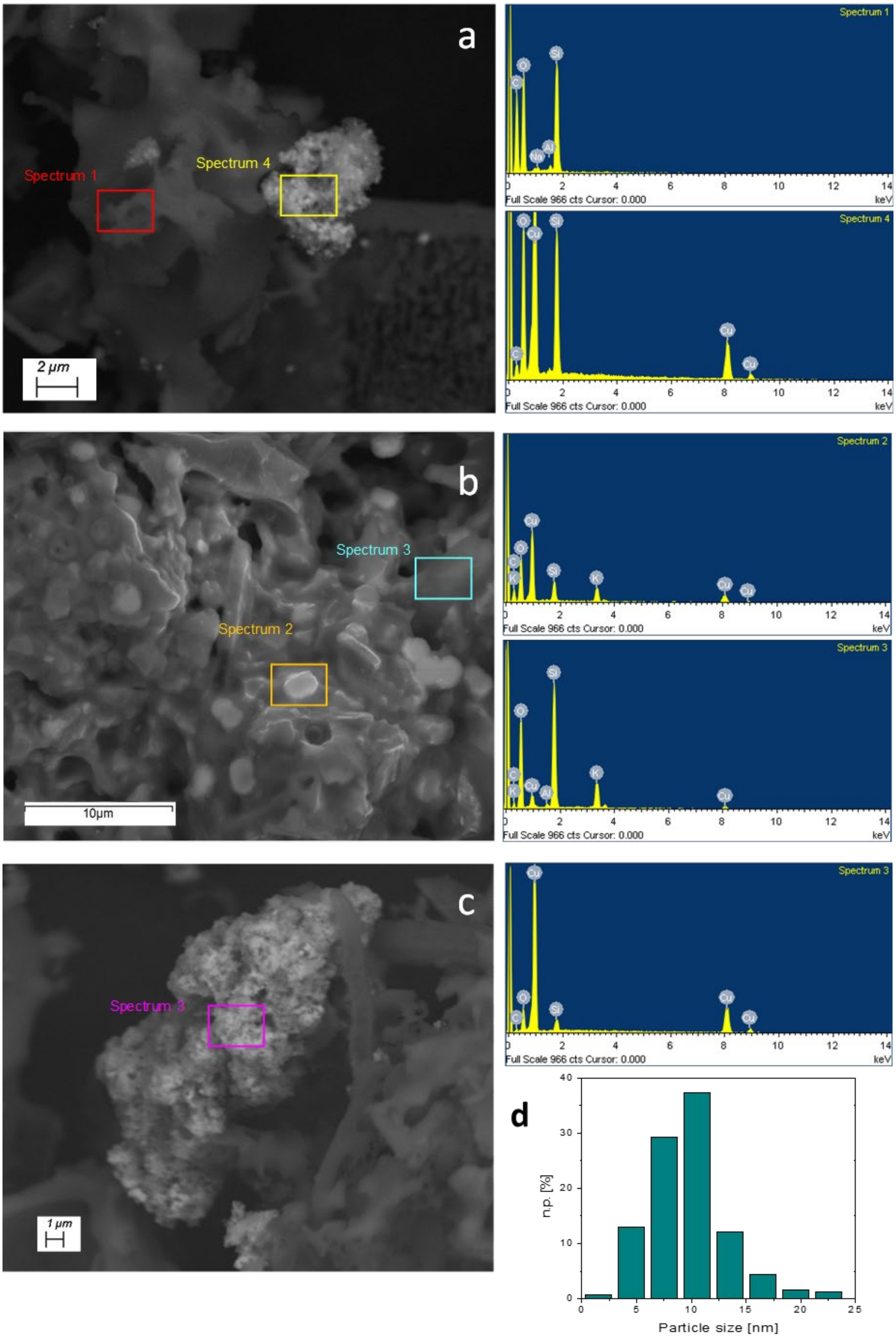

Figure S9: SEM images of the CuNPs/Celite before (a) and after reaction (b) and after reactivation (c). Images acquired in BSE mode in which the CuNPs appear brighter with respect to the support, EHT= 15.00 kV. Instrumental magnification 7300×(a) and 8000× (b and c);(d) Particle size distribution of the CuNPs/celite catalyst (n=246). Histogram of the Cu particle size distribution was obtained by HR-TEM.

5.1 Specific Surface Area and of number of exposed Cu site on the catalyst surface.

Based on the particle size distribution, the corresponding metal Specific Surface Area (SSA, m^2/g) of the Cu nanoparticles (supposed to be spherical) can be calculated by the equation:

$3\Sigma n_i r_i^2/(\delta_{Cu}\Sigma n_i r_i^3)$ m^2/g (r_i is the mean radius of the size class containing n_i particles, and δ_{Cu} the volumetric mass of Cu, equal to 8,96 g/cm^3).

SSA= 269.896 m^2/g

269.896 $m^2/g \times$ 63,546 g/mol= 17150.8 m^2/mol_{Cu}

6.022×10^{23} at/mol : 17150.8 m^2/mol= 3.511×10^{19} at/m^2

500 mg of catalyst contain 5 wt% Cu (from ICP), therefore 25 mg Cu: (269.896: 1000) × 25= 6,7474 m^2

3.511×10^{19} at/m^2 × 6,7474 m^2= $2,36 \times 10^{20}$ exposed Cu atoms.

6. Batch synthesis of 2-(2-aminophenyl)-1-(pyrrolidin-1-yl)ethan-1-one: stability and purity assessment

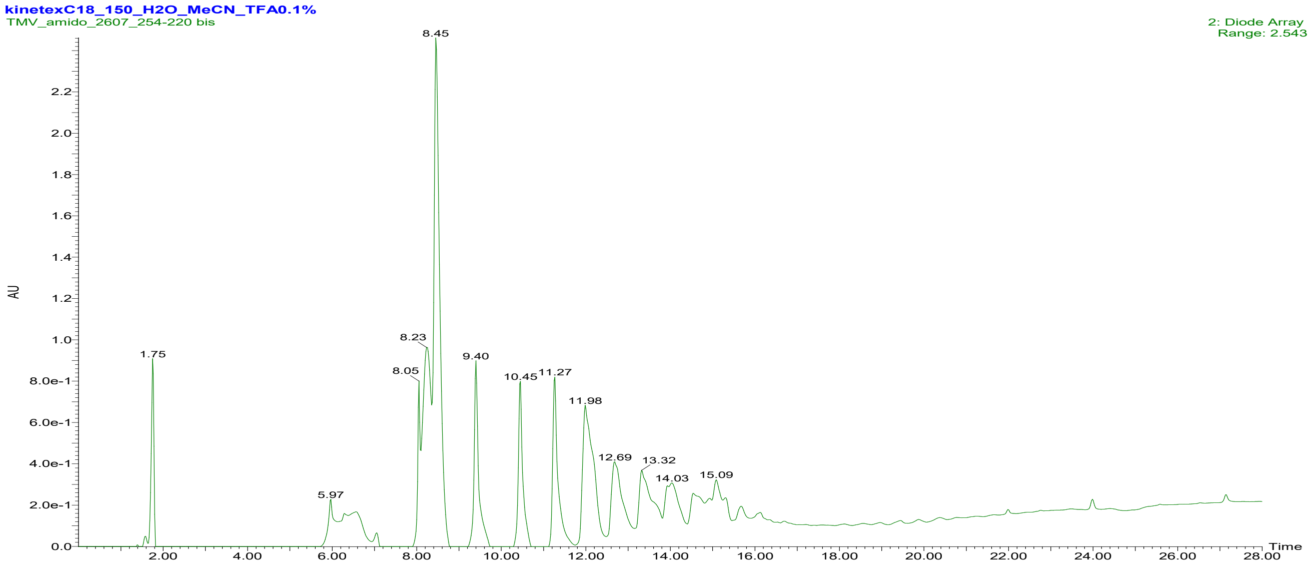

Figure S10 *HPLC Chromatogram at 220 nm of 2-(2aminophenyl)-1-(pyrrolidin-1-yl)ethan-1-one TH to obtain 2-(2-aminophenyl)-1-(pyrrolidin-1-yl)ethan-1-one (7) in oil bath. Reaction condition: nitrobenzene derivative (1 eq), KOH (2 eq), catalyst (20 mol%), EG; T: 130 ºC, 1.5 hr.*

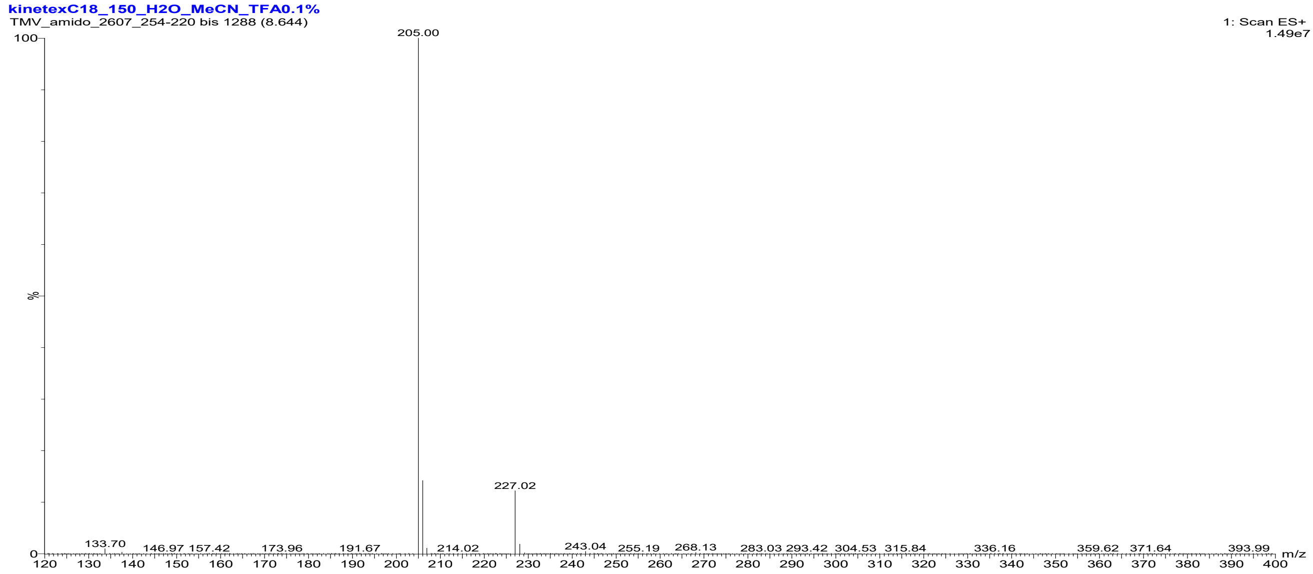

Figure S11. *MS Profile of 2-(2-aminophenyl)-1-(pyrrolidin-1-yl)ethan-1-one (7) r.t 8,644 min (See chromatogram above). C.I. MW 204,27 found $[M+H]^+$ 205,00; $[M+Na]^+$227,02*

7. E-factor Analysis

E-factor was calculated with the general equation:

$$E\,factor = \frac{Kg\ of\ waste}{Kg\ of\ product}$$

3.1 E-factor calculation and distribution analysis for literature flow protocols 1:

S. Doherty, J. G. Knight, T. Backhouse, A. Bradford, F. Saunders, R. A. Bourne, T. W. Chamberlain, R. Stones, A. Clayton and K. Lovelock, *Catal. Sci. Technol.*, 2018,8, 1454-1467

Reactant:

nitrobenzene: 6,1555 g

sodium borohydride: 4,7288 g

Reaction Solvent and Auxiliaries:

Sodium hydroxide: 9.999g

water: 250 g

ethanol: 197 g

Catalyst:

Supported Pd catalyst : 0.092 g

Sand= 3.9 g

Work-up materials:

Not described

Product:

Yield 99%: 4.9 g (Yields determined by ^{1}H NMR spectroscopy using dioxane as internal standard)

E-factor: $\{[6.155 + 4.7288]_{[reactants]}\ [9.999]_{[auxiliaries]} + [0.092 + 3.9]_{[catalyst]} + [250 + 197]_{[solvents]} - 4.9_{[product]}\} / 4.9 =$ **100,35**

Raw E-factor profile

E kernel	1,36
E excess	0,61
E aux	99,13
E catalyst	0,87
E workup and purification	-
E total reaction	100,35

Ai, Y., Hu, Z., Shao, Z. et al. Egg-like magnetically immobilized nanospheres. *Nano Res.* 2018, 11, 287–299.

Reactant:

Nitrophenol: 5,560 g

Hydrazine hydrate: 4,0 g

Reaction Solvent:

Methanol: 63,304 g

Catalyst:

Supported $Fe_2O_3{\cdot}xBi_2O$ catalyst : 0650 g

Silica= 1,2 g

Work-up materials:

Not described

Product:

Amino phenol: 96% 4,190 g

E-factor: $\{[5,560 + 4,0]_{[reactants]} + [0.650 + 1,2]_{[catalyst]} + [63,304]_{[solvents]} - 4.190_{[product]}\} / 4,190 =$ **20,81**

Raw E-factor profile

E kernel	1,28
E excess	0,36
E aux	19,09
E catalyst	0,45
E workup and purification	.
E total reaction	20,81623

Shen, M.; Bendel, C.; Vibbert, H. B.; Khine, P. T.; Norton, J. R.; Moment, A. J. *Green Chem.* **2023**, 25 (18), 7183-7188.

Reactant:

Nitrobenzene: 1,230 g

Ammonia Borane: 0,321 g

Reaction Solvent:

Methanol: 31,4 g

Catalyst:

Pd/C: 0,080

Work-up materials:

Not described

Product:

Aniline: 99% 0,921 g

E-factor: $\{[1,23 + 0,321]_{[reactants]} + [0.08]_{[catalyst]} + [31,4]_{[solvents]} - 0,921_{[product]}\} / 0,021 =$ **34,86**

Raw E-factor profile

E kernel	0,68
E excess	-
E aux	34,09
E catalyst	0,08
E workup and purification	.
E total reaction	34,86

This work

Reactant:

Nitrobenzene: 16.004 g

Ethylene glycol 8,0691 g

Reaction Solvent and Auxiliary:

Potassium hydroxide: 14.586 g

Ethylene glycol: 280,5 g (recovered 75%, 210,37 g)

Catalyst:

Cu catalyst : 0.5 g

Work-up and purification:

DOWEX Resin 52 g (recovered 99%, 5 1,48 g)

Methanolic/NH_3 103 g

Ethanol 205 (recovered 90% 184 g)

Product:

1 1,1 g Yield 93%: 4.9 g

E-factor: {[16,004 + 8,0691]$_{[reactants]}$ + [14,586]$_{[auxiliary]}$ + [0.5] $_{[catalyst]}$+ [280,5]$_{[solvents]}$ + [52 +103 + 205]$_{[work-up]}$ –1 1,1$_{[product]}$ – 210,37$_{[EG\ recovered]}$ – 5 1,48 $_{[resin\ recovered]}$} – 184 $_{[EtOH\ recovered]}$ / 1 1,9 = **20,19**

Raw E-factor profile

E kernel	1,16
E excess	-
E aux reaction	7,63
E catalyst	0,04
E purification	11,16
E aux reaction+workup	18,71
E total reaction total	8,30
E total reaction and purification	20,19

8. NMR Spectra of intermediate and final compounds

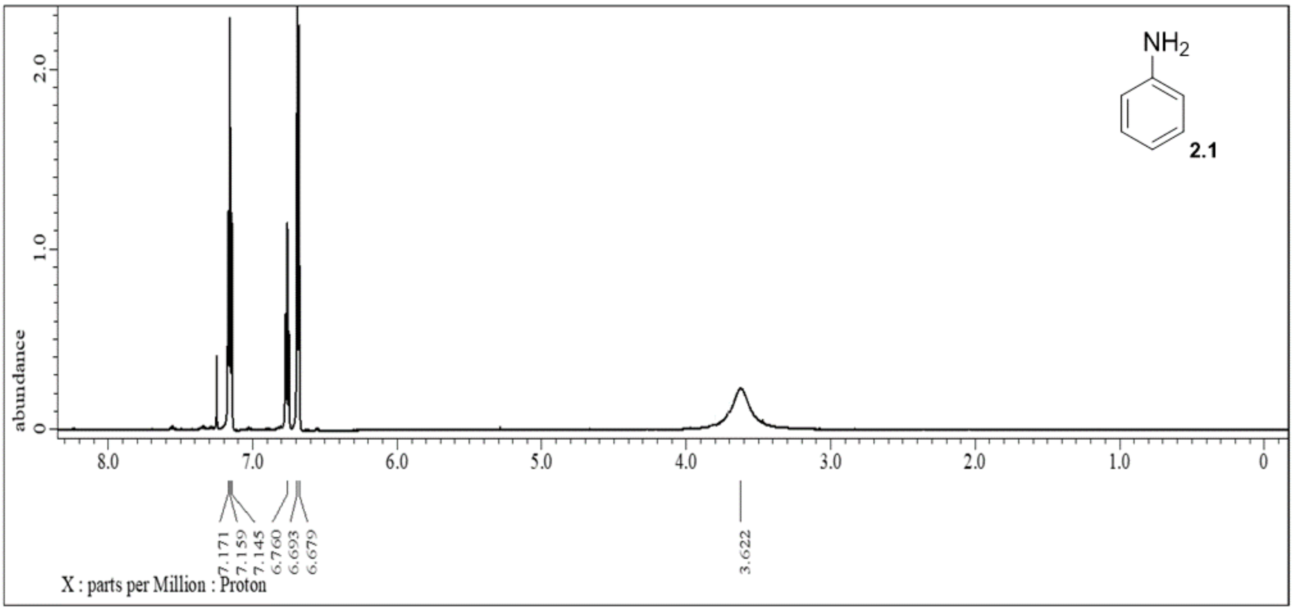

Figure S12. 1H NMR (600 MHz, $CDCl_3$) of Aniline (1)

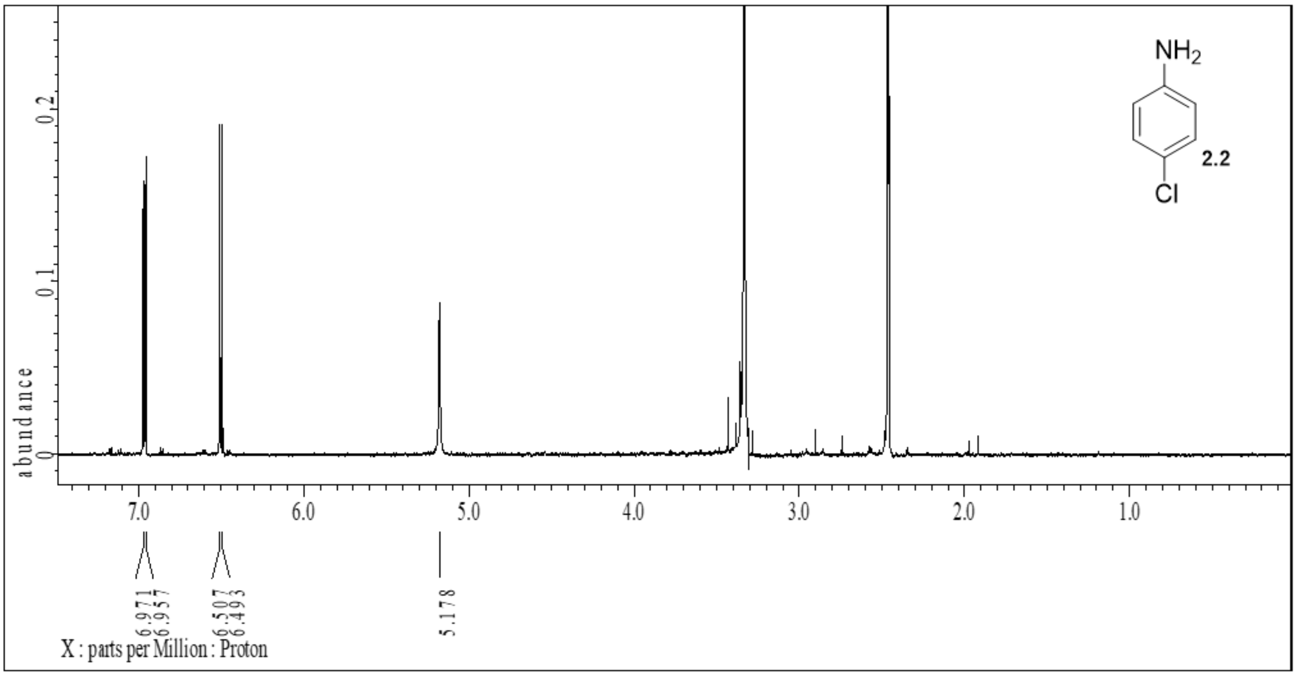

Figure S13. ^1H NMR (300 MHz, DMSO-d6) of p-Chloroaniline (2)

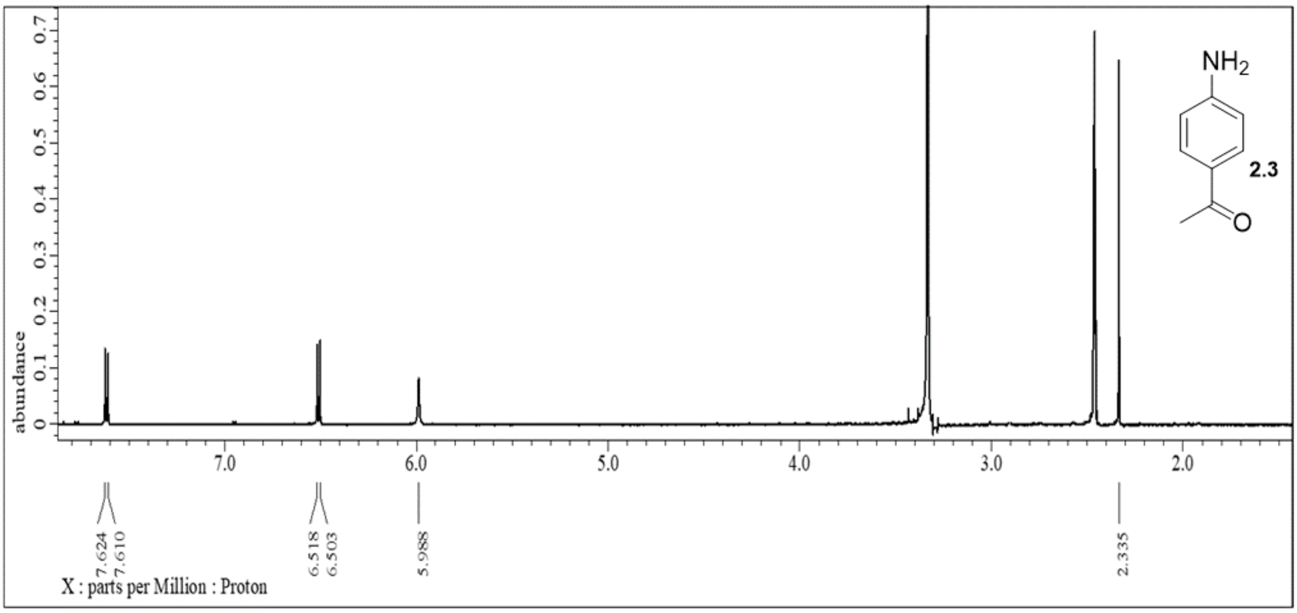

Figure S14. 1H NMR (600 MHz, DMSO-d6) of p-Aminoacetophenone (3):

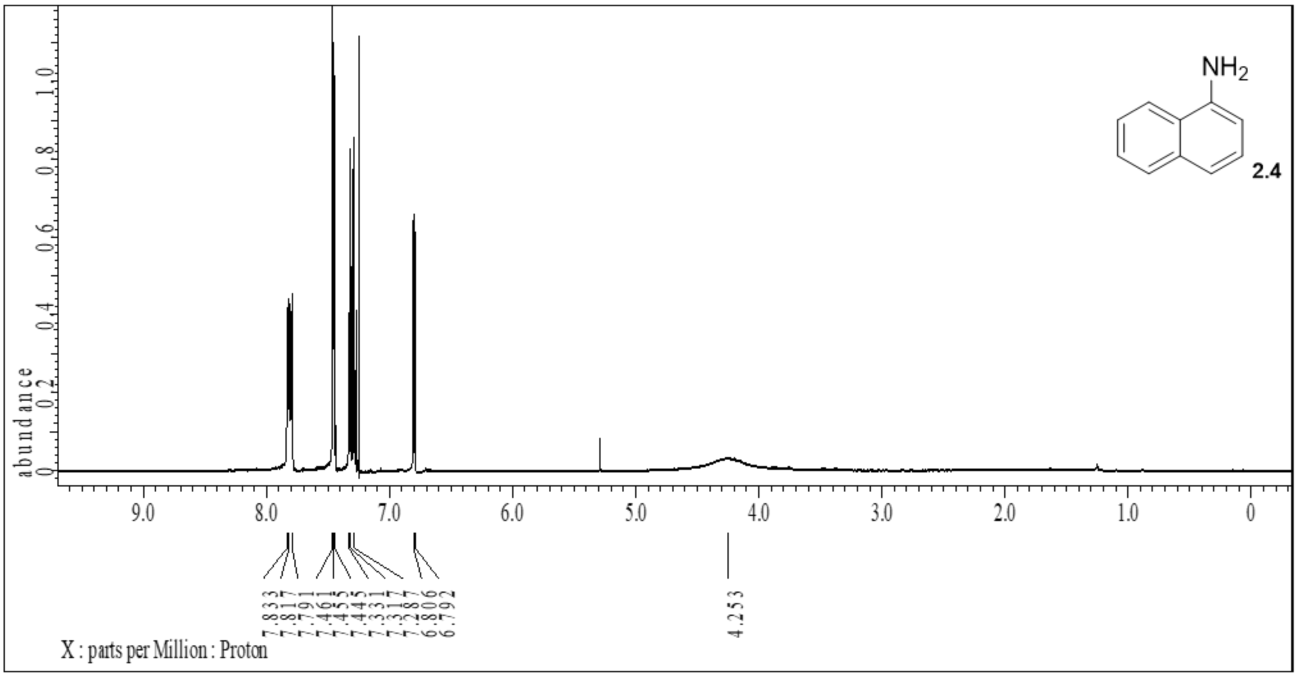

Figure S15. ^{1}H NMR (600 MHz, $CDCl_3$) of 1-naphthylamine (4):

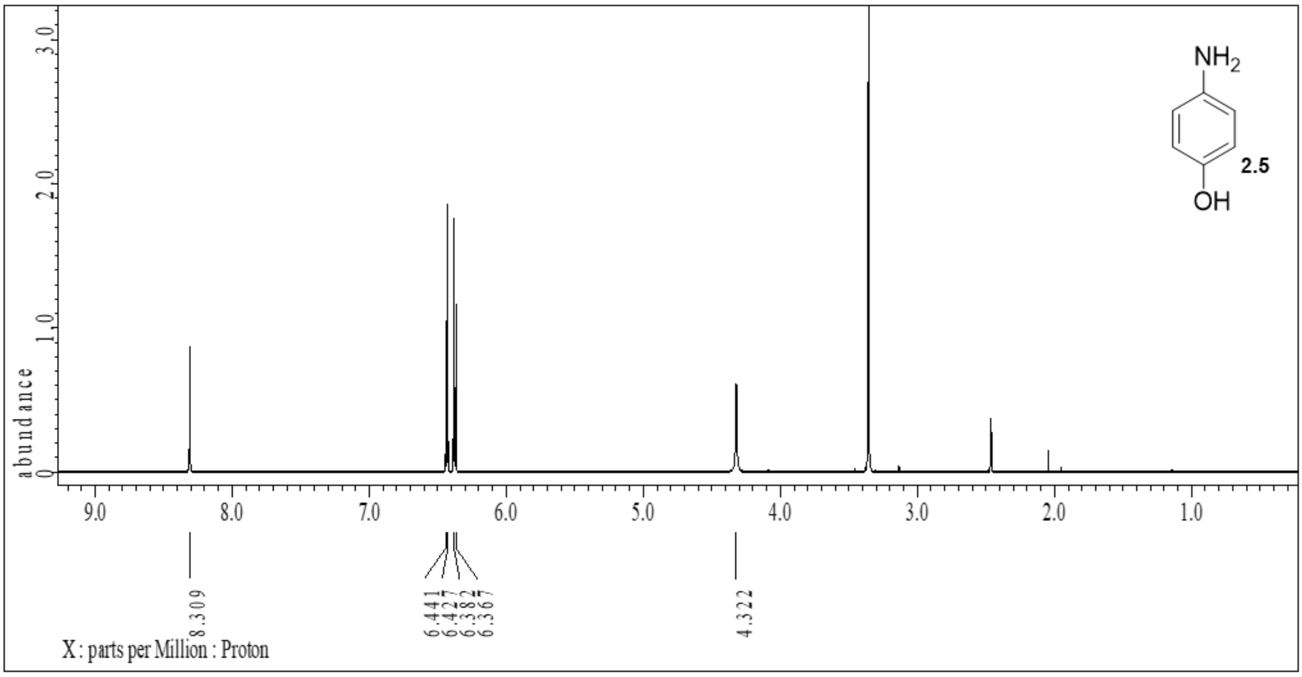

Figure S16. ^{1}H NMR (600 MHz, DMSO-d6) of p-Aminophenol (5)

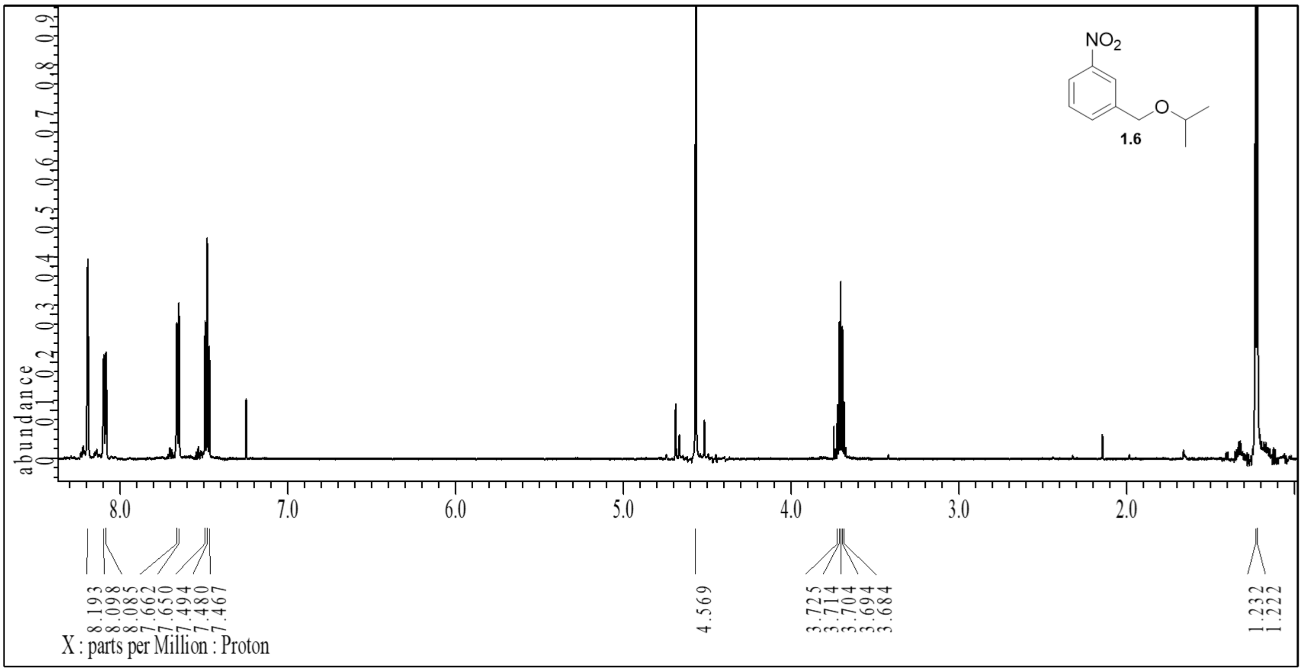

Figure S17. ^{1}H NMR (600 MHz, CDCl3) of 1-(isopropoxymethyl)-3-nitrobenzene (1,6)

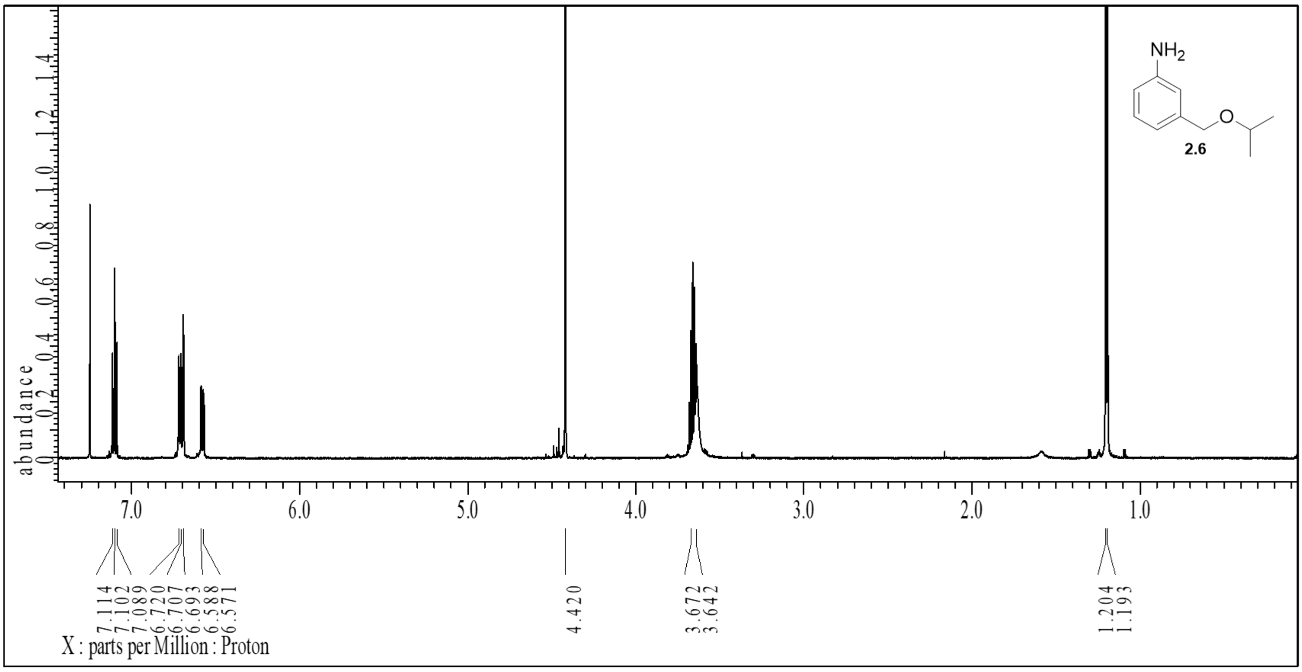

Figure S18. ^{1}H NMR (600 MHz, $CDCl_3$) of 3-(isopropoxymethyl)aniline (6)

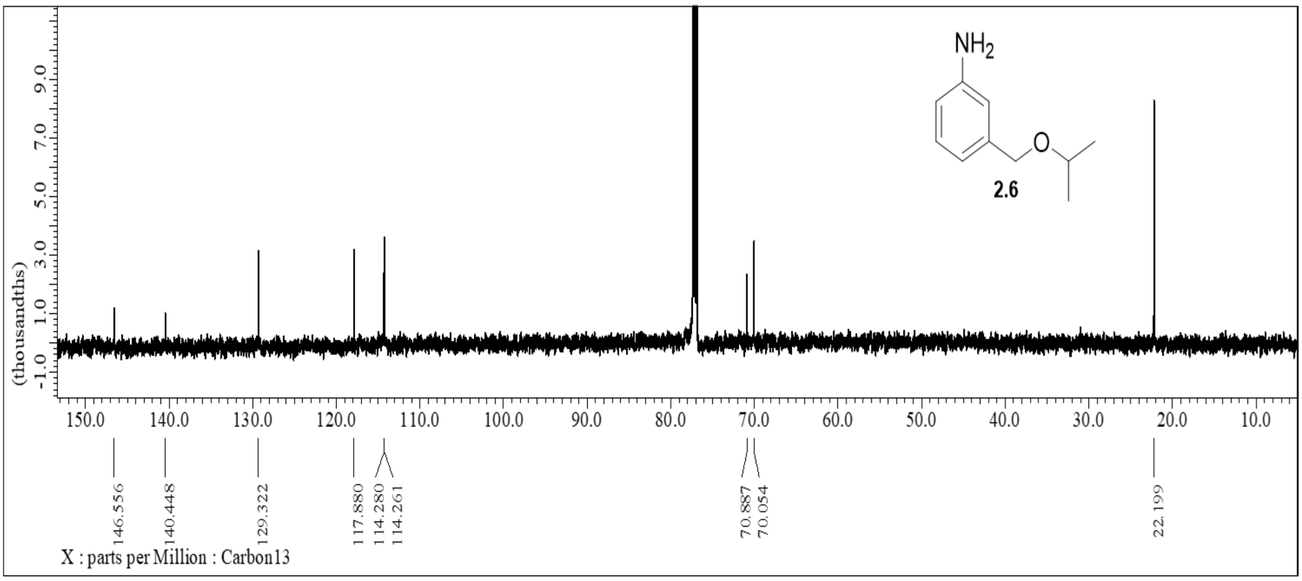

Figure S19. ^{13}C NMR (600 MHz, $CDCl_3$) of 3-(isopropoxymethyl)aniline (6)

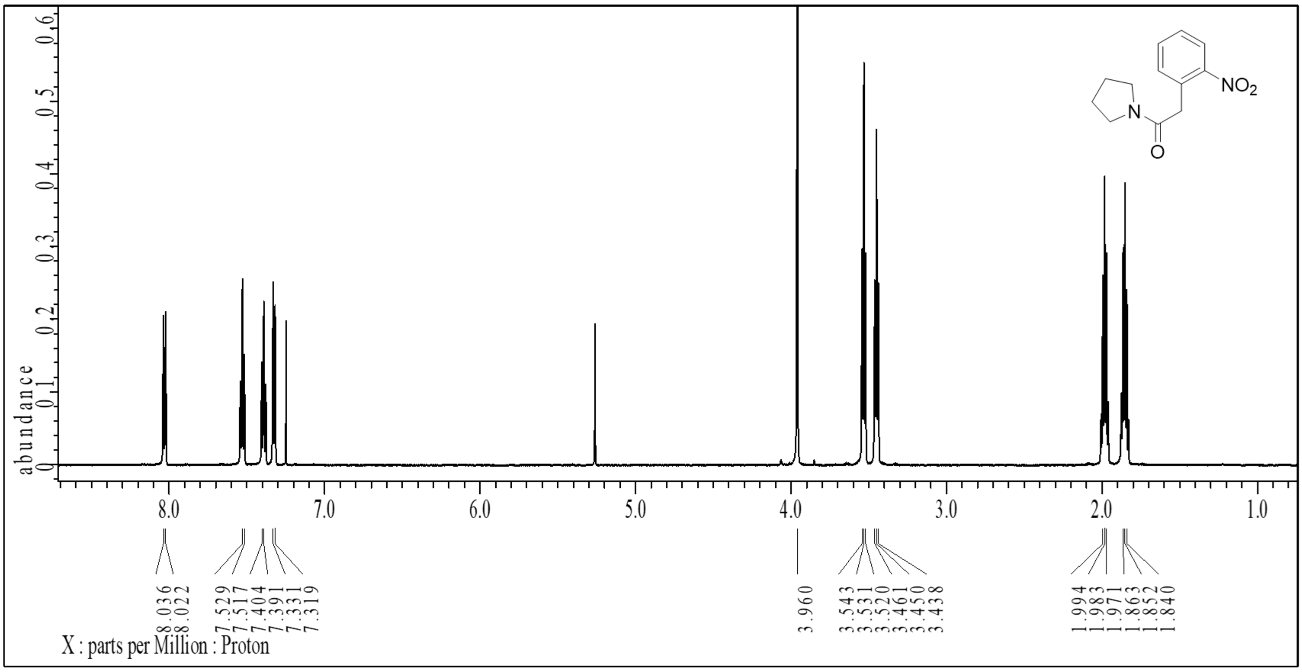

Figure S20. ***^{1}H NMR (600 MHz, $CDCl_3$) of 2-(2-nitrophenyl)-1-(pyrrolidin-1-yl)ethan-1-one (1,7)***

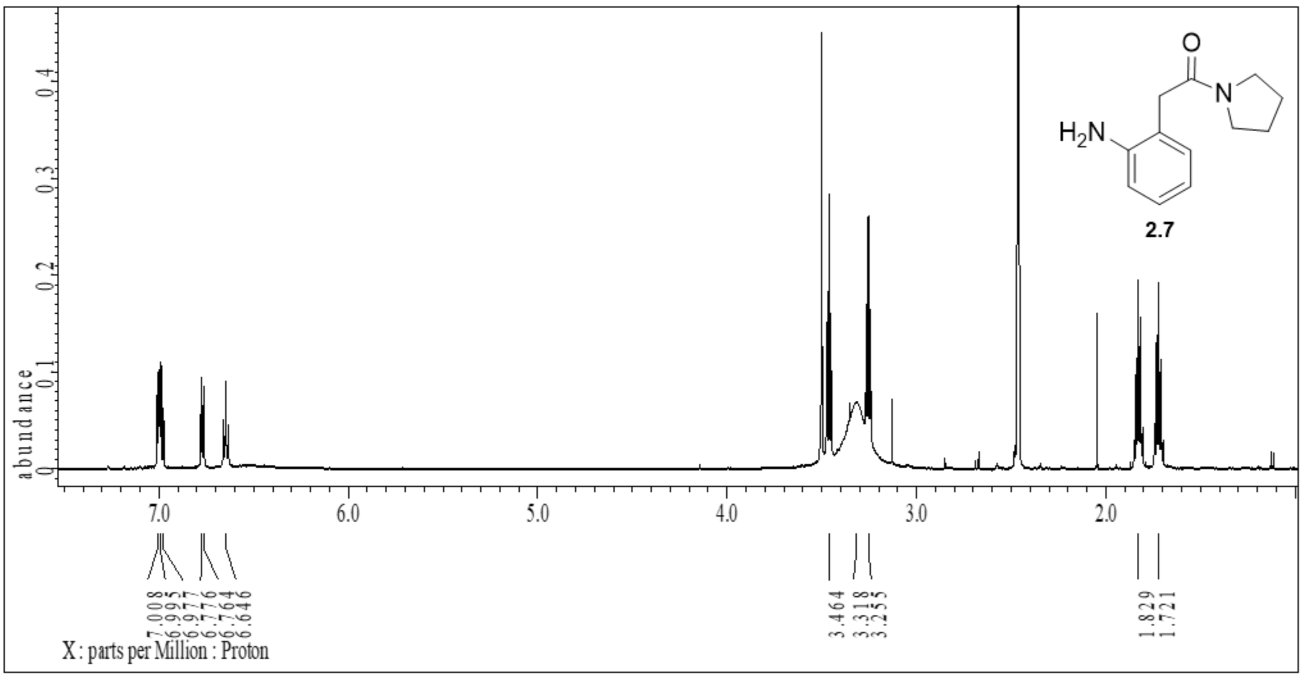

Figure S21. ^{1}H NMR (600 MHz, DMSO-d6) of 2-(2-aminophenyl)-1-(pyrrolidin-1-yl)ethan-1-one (7)

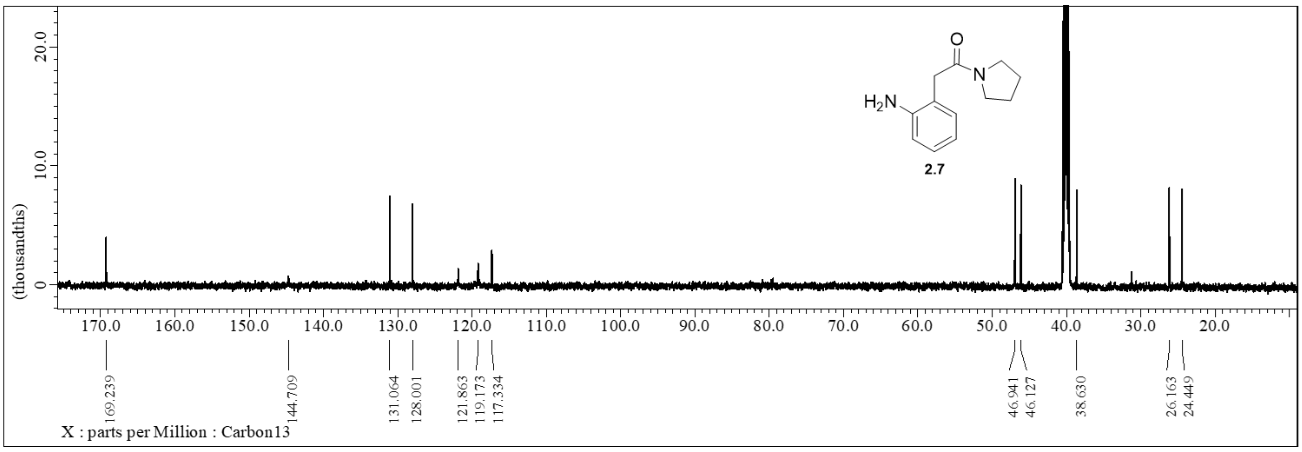

Figure S22. ^{13}C NMR (150 MHz, DMSO-d6) of 2-(2-aminophenyl)-1-(pyrrolidin-1-yl)ethan-1-one (7)

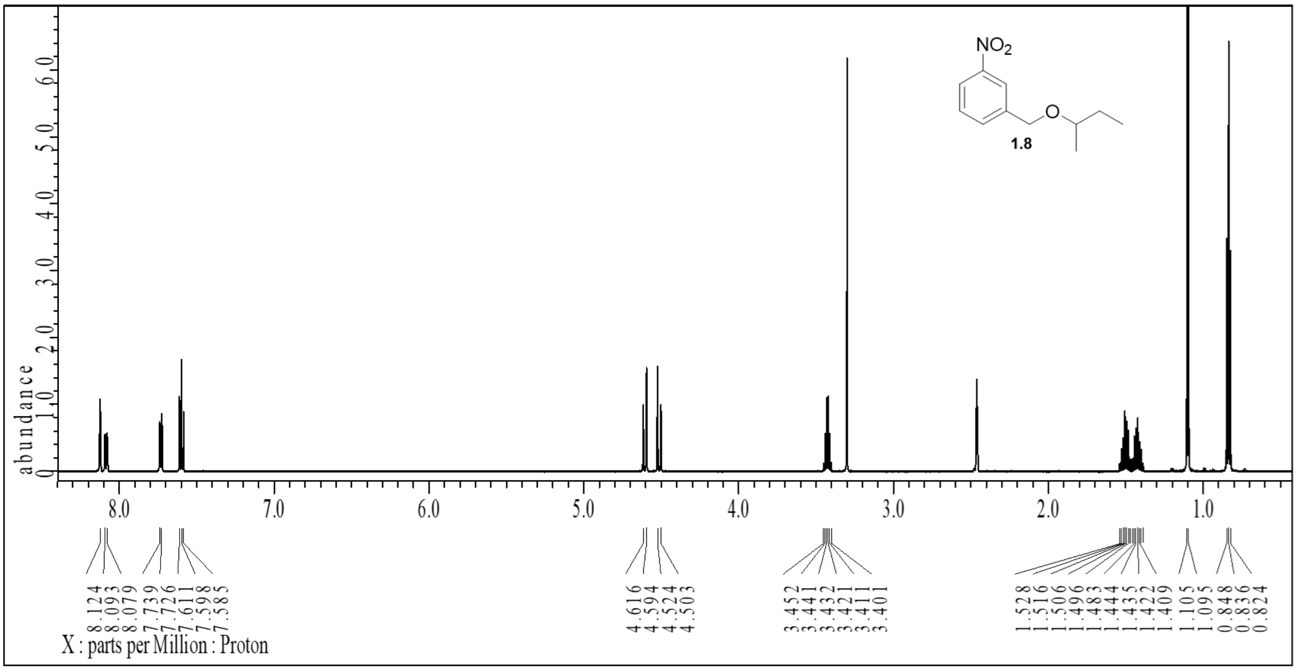

Figure S23 ^{1}H NMR (600 MHz, DMSO-d6) of 1-(sec-butoxymethyl)-3-nitrobenzene (1.8)

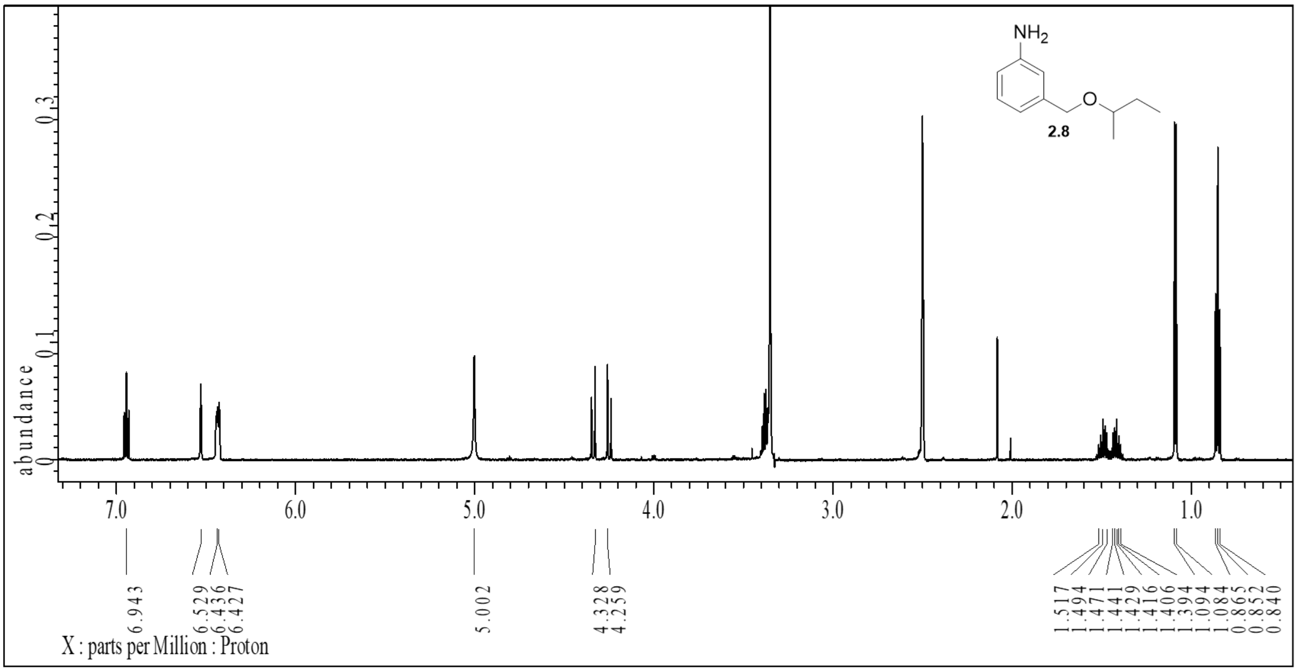

Figure S24. 1H NMR (600 MHz, DMSO-d6) of 3-(sec-butoxymethyl)aniline (8)

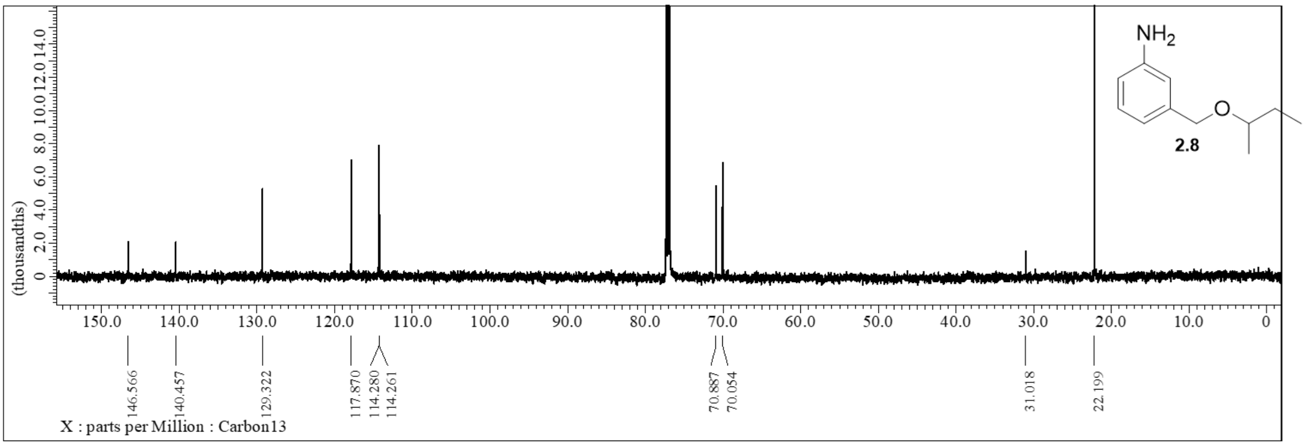

Figure S25. ^{1}H NMR (150 MHz, DMSO-d6) of 3-(sec-butoxymethyl)aniline (8)

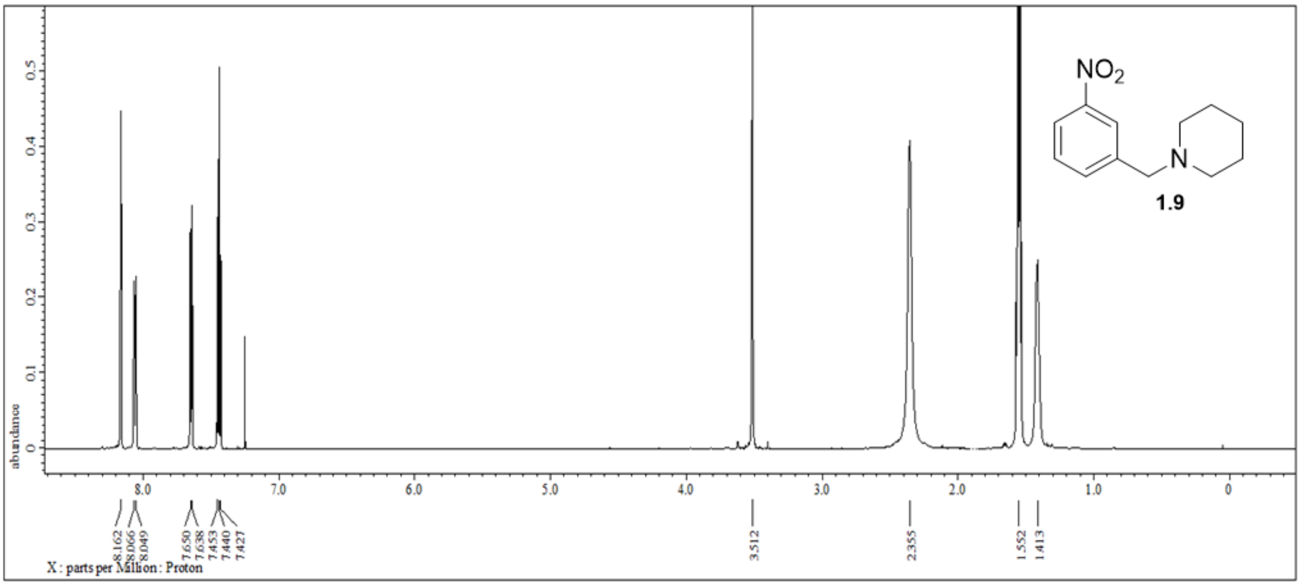

Figure S26. ***^{1}H NMR (600 MHz, CDCl3) of 1-(3-nitrobenzyl)piperidine (1.9)***

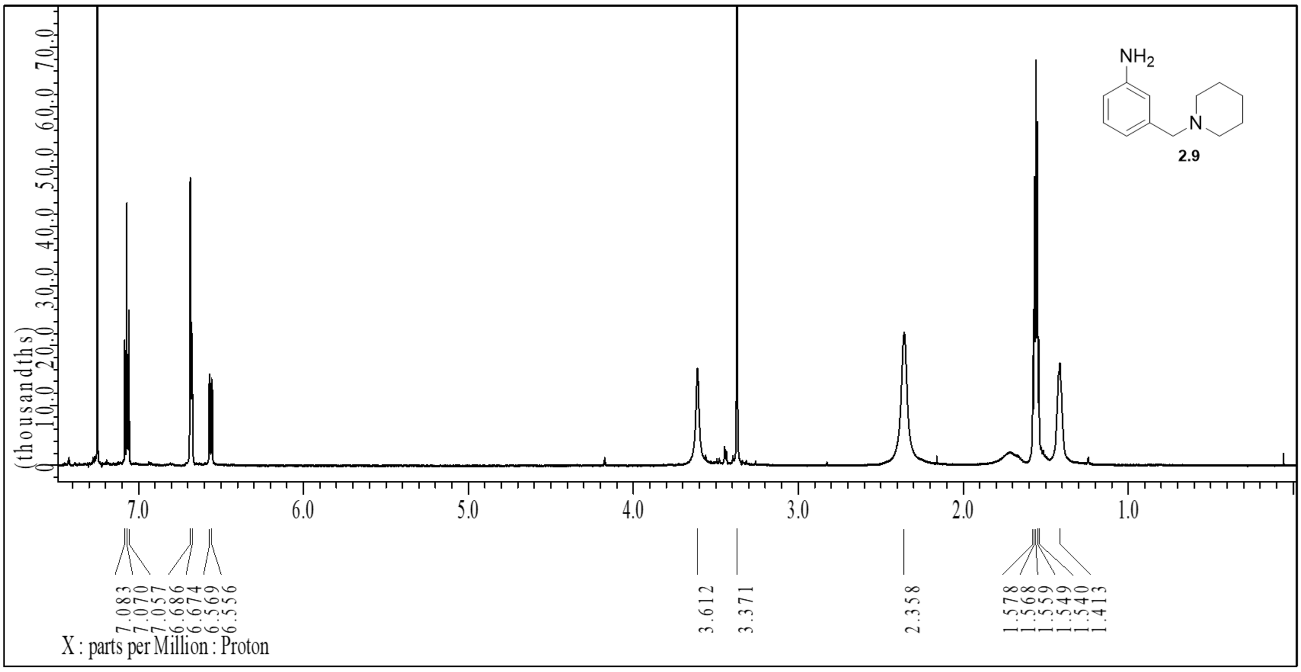

Figure S27. ***^{1}H NMR (600 MHz, CDCl3) of 3-(piperidin-1-ylmethyl)aniline (9)***

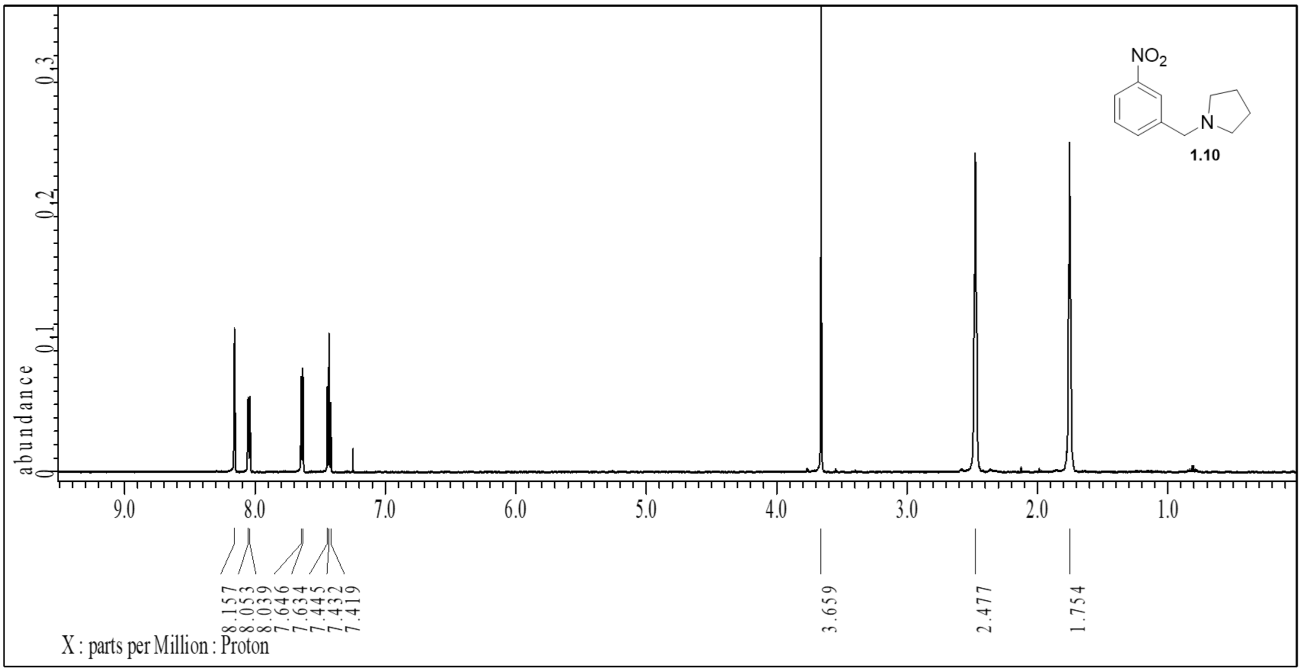

Figure S28. ¹H NMR (600 MHz, CDCl3) of 1-(3-nitrobenzyl)pyrrolidine (1.10)

)

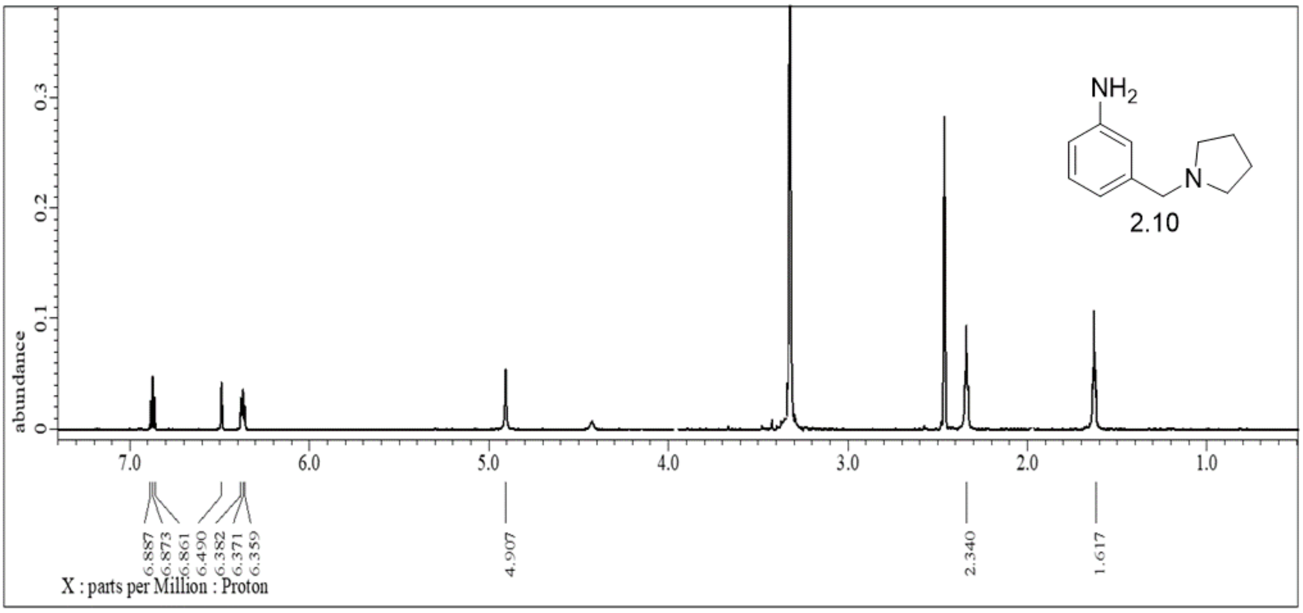

Figure S29. ¹H NMR (600 MHz, CDCl3) of 3-(pyrrolidin-1-ylmethyl)aniline (10)

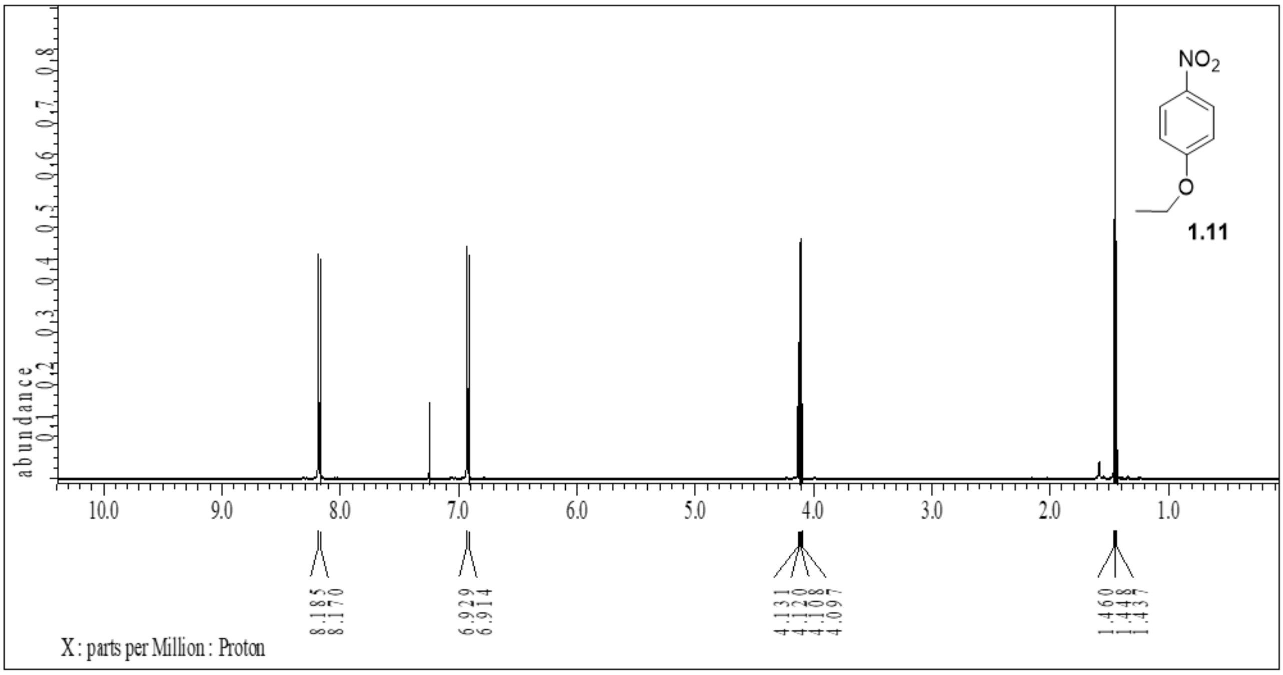

Figure S30. ^{1}H NMR (600 MHz, CDCl3) of 4-etoxy aniline (1.11)

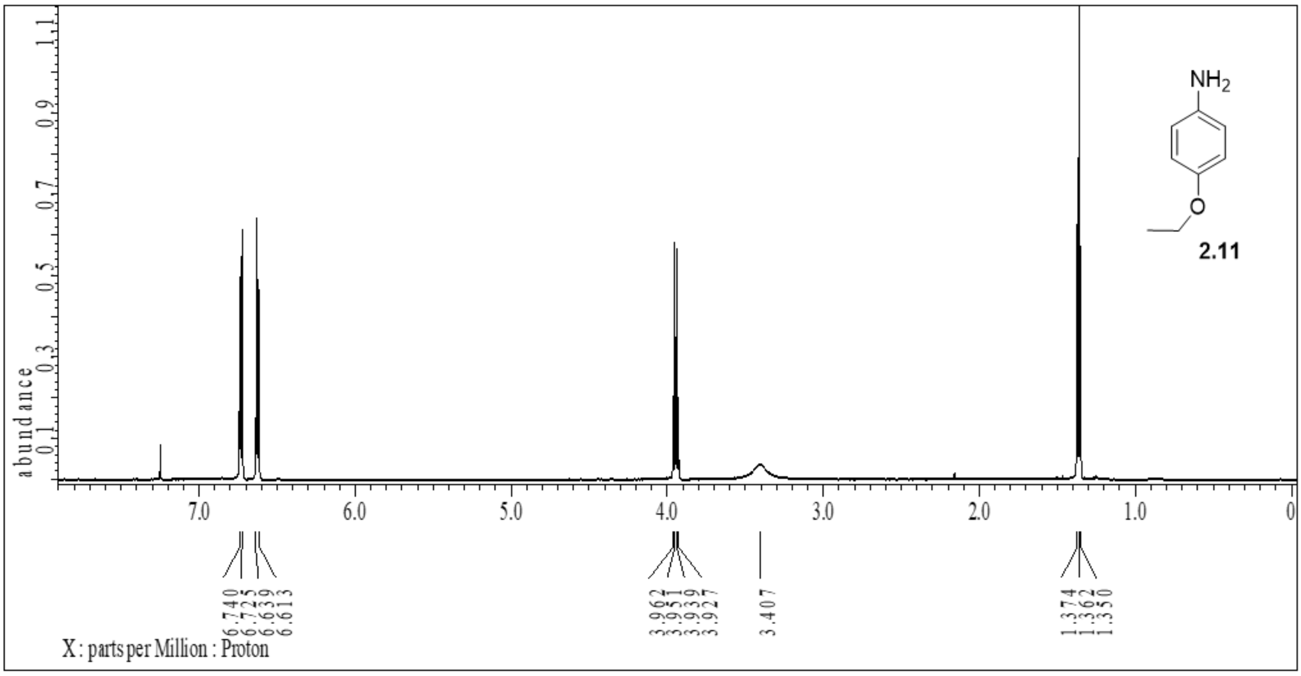

Figure S31. ^{1}H NMR (600 MHz, $CDCl_3$) of 4-etoxy-aniline (11)

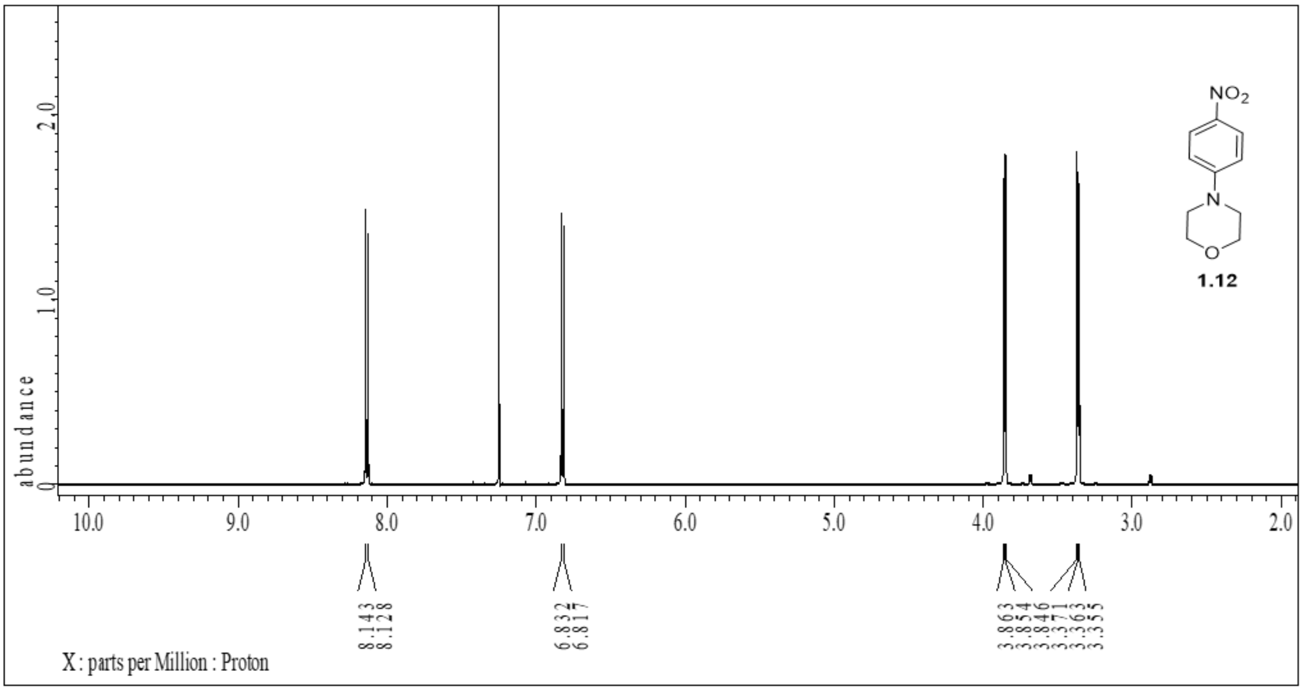

Figure S32. ¹H NMR (600 MHz, CDCl3) of 4-nitrophenyl morpholine (1.12)

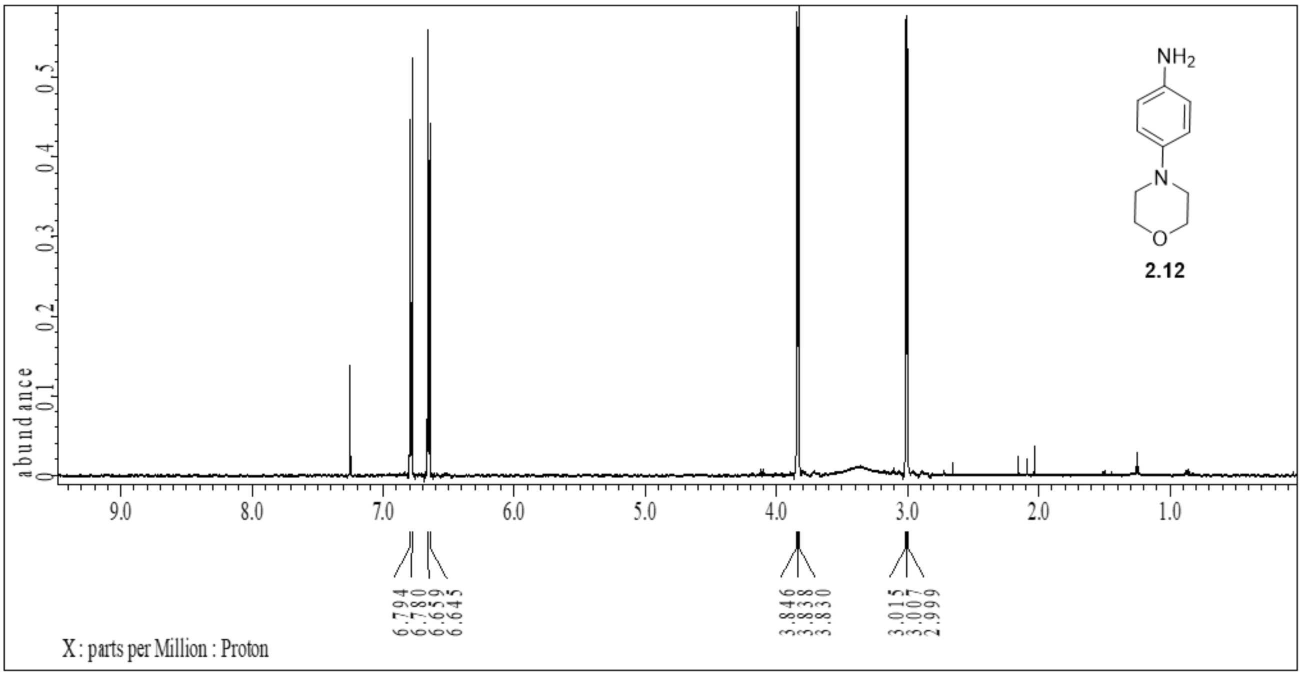

Figure S33. ¹H NMR (600 MHz, CDCl3) of 4-morpholino aniline (2.12)

9. References

(1) Bunce, R. A.; Easton, K. M. A Versatile and convenient synthesis of alkyl nitroaromatic ethers by nucleophilic aromatic substitution. *Org. Prep. Proced. Int.* **2004**, *36* (1), 76-81.

(2) Huh, D. H.; Jeong, J. S.; Lee, H. B.; Ryu, H.; Kim, Y. G. An efficient method for one-carbon elongation of aryl aldehydes via their dibromoalkene derivatives. *Tetrahedron* **2002**, *58* (50), 9925-9932.

(3) Zuo, S.-J.; Zhang, S.; Mao, S.; Xie, X.-X.; Xiao, X.; Xin, M.-H.; Xuan, W.; He, Y.-Y.; Cao, Y.-X.; Zhang, S.-Q. Combination of 4-anilinoquinazoline, arylurea and tertiary amine moiety to discover novel anticancer agents. *Bioorg. Med. Chem.* **2016**, *24* (2), 179-190.

(4) Wubbels, G. G.; Danial, H.; Policarpio, D. Temperature Dependence of Regioselectivity in Nucleophilic Photosubstitution of 4-Nitroanisole. The Activation Energy Criterion for Regioselectivity. . *J.l Org. Chem.* **2010**, *75* (22), 7726-7733.

(5) Liu, C.; Yu, T.; Yang, T.; Sun, H.; Qin, C.; Jia, Q.; Chu, C. Facile Preparation of 4-(4-Nitrophenyl)morpholin-3-one via the Acid-Catalyzed Selective Oxidation of 4-(4-Nitrophenyl)morpholine by Sodium Chlorite as the Sole Oxidant. *Org. Proc. Res. Develop.* **2020**, *24* (11), 2633-2638.

(6) Potdar, A.; Protasova, L. N.; Thomassen, L.; Kuhn, S. Designed porous milli-scale reactors with enhanced interfacial mass transfer in two-phase flows. *React.Chem, Eng,* **2017**, *2* (2), 137-148.

(7) Kreye, O.; Wald, S.; Meier, M. A. R. Introducing Catalytic Lossen Rearrangements: Sustainable Access to Carbamates and Amines. *Adv. Synth. Catal.* **2013**, *355* (1), 81-86.

(8) Sharma, U.; Kumar, P.; Kumar, N.; Kumar, V.; Singh, B. Highly Chemo- and Regioselective Reduction of Aromatic Nitro Compounds Catalyzed by Recyclable Copper(II) as well as Cobalt(II) Phthalocyanines. *Adv. Synth. Catal.* **2010**, *352* (11-12), 1834-1840.

(9) Zhang, T.; Zhang, Y.; Zhang, W.; Luo, M. A Convenient and General Reduction of Amides to Amines with Low-Valent Titanium. *Adv. Synth. Catal.s* **2013**, *355* (14-15), 2775-2780.

(10) Gonec, T.; Zadrazilova, I.; Nevin, E.; Kauerova, T.; Pesko, M.; Kos, J.; Oravec, M.; Kollar, P.; Coffey, A.; Mahony, J.; et al. Synthesis and Biological Evaluation of N-Alkoxyphenyl-3-hydroxynaphthalene-2-carboxanilides. In *Molecules*, 2015; Vol. 20, pp 9767-9787.

(11) Gao, J.; Bhunia, S.; Wang, K.; Gan, L.; Xia, S.; Ma, D. Discovery of N-(Naphthalen-1-yl)-N′-alkyl Oxalamide Ligands Enables Cu-Catalyzed Aryl Amination with High Turnovers. *Org. Lett.* **2017**, *19* (11), 2809-2812.
